# Increased Transcript Complexity in Genes Associated with Chronic Obstructive Pulmonary Disease

**DOI:** 10.1371/journal.pone.0140885

**Published:** 2015-10-19

**Authors:** Lela Lackey, Evonne McArthur, Alain Laederach

**Affiliations:** Department of Biology, University of North Carolina, Chapel Hill, North Carolina, United States of America; Helmholtz Zentrum München, GERMANY

## Abstract

Genome-wide association studies aim to correlate genotype with phenotype. Many common diseases including Type II diabetes, Alzheimer’s, Parkinson’s and Chronic Obstructive Pulmonary Disease (COPD) are complex genetic traits with hundreds of different loci that are associated with varied disease risk. Identifying common features in the genes associated with each disease remains a challenge. Furthermore, the role of post-transcriptional regulation, and in particular alternative splicing, is still poorly understood in most multigenic diseases. We therefore compiled comprehensive lists of genes associated with Type II diabetes, Alzheimer’s, Parkinson’s and COPD in an attempt to identify common features of their corresponding mRNA transcripts within each gene set. The *SERPINA1* gene is a well-recognized genetic risk factor of COPD and it produces 11 transcript variants, which is exceptional for a human gene. This led us to hypothesize that other genes associated with COPD, and complex disorders in general, are highly transcriptionally diverse. We found that COPD-associated genes have a statistically significant enrichment in transcript complexity stemming from a disproportionately high level of alternative splicing, however, Type II Diabetes, Alzheimer’s and Parkinson’s disease genes were not significantly enriched. We also identified a subset of transcriptionally complex COPD-associated genes (~40%) that are differentially expressed between mild, moderate and severe COPD. Although the genes associated with other lung diseases are not extensively documented, we found preliminary data that idiopathic pulmonary disease genes, but not cystic fibrosis modulators, are also more transcriptionally complex. Interestingly, complex COPD transcripts are more often the product of alternative acceptor site usage. To verify the biological importance of these alternative transcripts, we used RNA-sequencing analyses to determine that COPD-associated genes are frequently expressed in lung and liver tissues and are regulated in a tissue-specific manner. Additionally, many complex COPD-associated genes are spliced differently between COPD and non-COPD patients. Our analysis therefore suggests that post-transcriptional regulation, particularly alternative splicing, is an important feature specific to COPD disease etiology that warrants further investigation.

## Introduction

Disease predisposition is likely driven by disturbances at multiple levels of gene regulation. Genetic variation affecting RNA in both the coding and non-coding portions of the genome has the potential to regulate transcript localization, degradation, splicing and translation, which contribute to disease [1–4]. A role for post-transcriptional regulatory processes in multigenic diseases is supported by the disproportionate number of single nucleotide polymorphisms (SNPs) mapping to non-coding regions of genes that are associated with disease [5]. However, the importance of post-transcriptional regulatory processes in multigenic disease etiologies has yet to be fully investigated. Therefore, we are interested in determining whether transcript complexity at gene loci could reveal underlying molecular mechanisms of specific multi-gene diseases.

**Fig 1:**
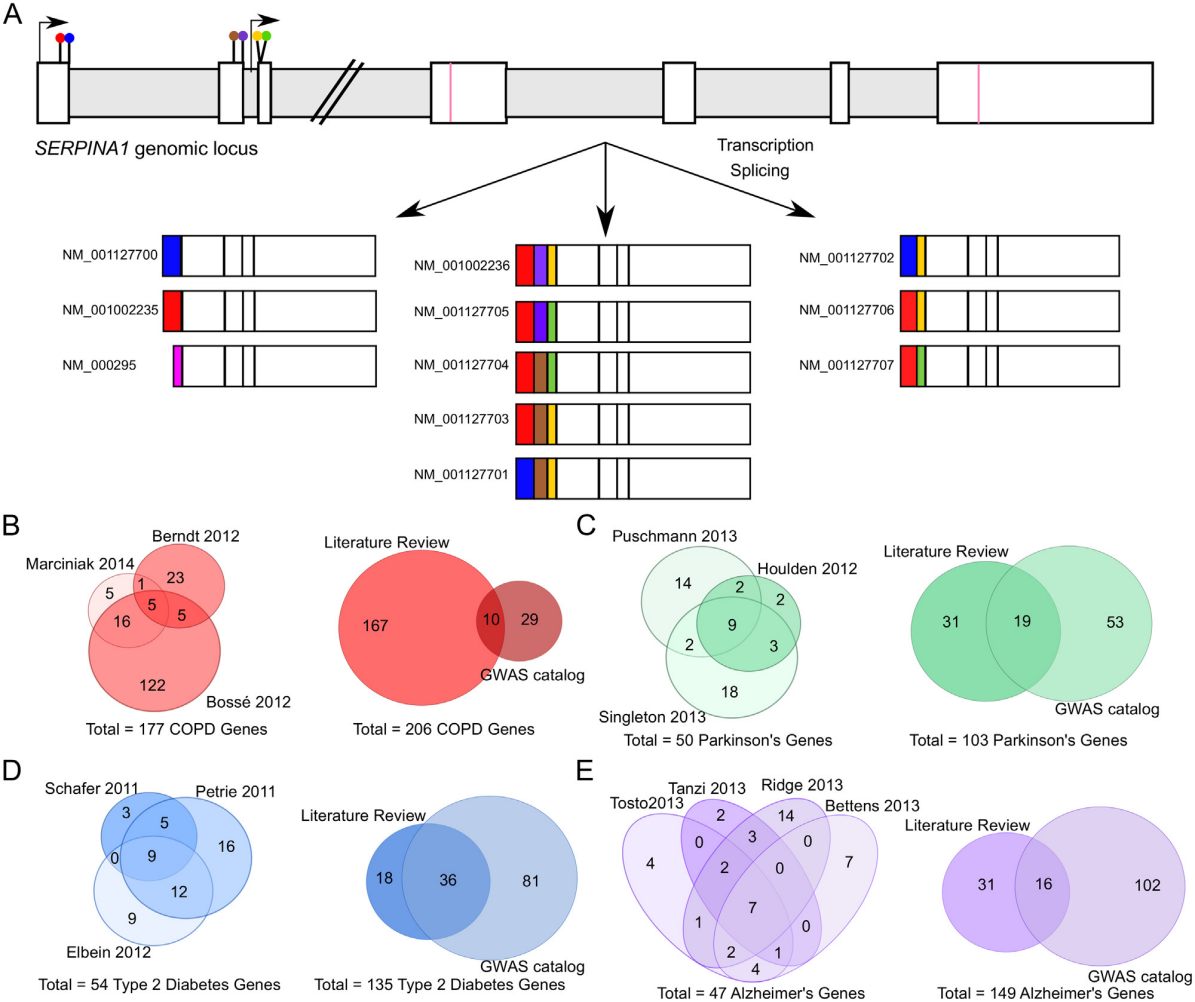
Combining sources to identify genes associated with COPD. *(A)* The COPD-associated gene, *SERPINA1*, is alternatively spliced to produce 11 different transcripts. Two transcription start sites are indicated by arrows; splice sites are shown as colored lollipops. Exons are indicated by white bars and introns by horizontal gray bars. The coding sequence start and stop codons are indicated with pink lines. 11 transcripts are depicted and colored by splice site selection. The 11 transcripts make *SERPINA1* a particularly complex gene in terms of alternative splicing, 99.5% of human genes have fewer transcripts. *(B)* COPD-associated genes were identified by merging disease-associated genes from different literature reviews (left) and combining them with genes from the NHGRI GWAS catalog (right). Other comparative disease lists were compiled in the same manner, including *(C)* Parkinson’s disease-associated genes, *(D)* Type 2 diabetes-associated genes and *(E)* Alzheimer’s disease-associated genes. The number of genes from each source is indicated in the Venn diagrams.

The basic premise for this study stems from a simple observation in the *SERPINA1* gene. This gene produces α-1-anti-trypsin, a protein that regulates the proteolytic activity of elastase, and broadly affects the inflammatory response in the lung [6, 7]. Individuals with severe α-1-anti-trypsin deficiency may develop COPD even if they are not smokers [8]. However, genetic disruptions to *SERPINA1* account for less than 5% of COPD cases [9]. Thus, α-1-anti-trypsin deficiency is predictive of COPD in only a small subset of individuals even among smokers. One particularly striking feature of SERPINA1 is its high transcriptional complexity—i.e. the gene yields eleven alternative transcripts (Fig 1A). Several different types of alternative splicing mechanisms generate this complexity of transcripts, including exon skipping and alternative 5’ and 3’ splice site usage. The gene is unusual in terms of its alternative splicing—in comparison, 95.5% of loci within a reference set of human genes annotated by RefSeq have less than five transcripts. Thus, *SERPINA1* is in the top 1% in terms of number of transcripts. In addition, *SERPINA1* harbors non-coding SNPs in its 5’ untranslated region (UTR) that are associated with COPD, suggesting post-transcriptional processes likely play a role in its regulation [10].

COPD is a lung disease defined as progressive airflow limitation [11]. It is the fourth leading cause of death in the world, and is a major burden for 64 million people globally [12]. Although smoking is a strong environmental factor for the development of COPD, whether or not an individual will develop the disease if he or she smokes is highly dependent on family history [9, 13, 14]. We investigate here whether the high transcriptional complexity of *SERPINA1* (Fig 1A) is a common feature of COPD associated genes and if high transcriptional complexity is a broader characteristic of other complex multigenic diseases.

## Results

### Identification of disease-associated gene loci

Since the cause of COPD can only be attributed to α-1-anti-trypsin deficiency in ~5% of patients, but many other genomic loci are associated with COPD development and progression, we manually curated a list of COPD associated genes to investigate their transcriptional complexity. First, we combined genes implicated in COPD from recent COPD literature reviews (Fig 1B, **left**) [14–17]. The subsequent list was merged with search results from the National Human Genome Research Institute (NHGRI) Genome Wide Association Study (GWAS) catalog to yield a total set of 206 putative COPD-associated gene loci (Fig 1B, **right**, and S1 Table) [18, 19]. Literature reviews contributed the majority of the gene list for COPD, as these represent the best form of manual curation for further analysis. We did not include the PubMed text-miner, Glad4U, and the SNP database, SNP4Disease in our gene list generation, although we did use these resources in a confirmatory manner (S1 Fig, S2 Table) [20, 21].

To assess transcript complexity in other complex diseases, we curated similar gene lists for Parkinson’s disease (PRK), Type 2 Diabetes (T2D) and Alzheimer’s disease (ALZ). These three diseases have different etiologies, but are also complex chronic diseases that include significant genetic components. We followed the same protocol to create lists of genes associated with PRK, T2D and ALZ from recent literature reviews and the NHGRI GWAS resource. We identified 103 PRK gene loci (Fig 1C, S3 Table) [18, 22–25] and 135 T2D gene loci (Fig 1D, S4 Table) [18, 26–29] as well as 149 ALZ gene loci (Fig 1E, S5 Table) [18, 30–35]. Interestingly, although all reviews were within similar time frames, the genes reported were not identical. While the reviews and the GWAS identified genes have more genes in common than would be expected randomly (S6, S7 and S8 Tables), they also each contribute unique genes for analysis. Our strategy of curating gene lists therefore guarantees broad pools of COPD, T2D, ALZ and PRK-associated genes to investigate alternative transcript usage.

### COPD-associated gene loci have a high level of transcriptional complexity

While a majority of gene loci (55.7%) have only one annotated RNA transcript (based on RefSeq annotation), others produce upwards of twenty or thirty different transcripts through alternative splicing and transcription start site usage. These transcripts include non-coding RNAs that regulate their own and other loci and alternatively spliced mRNAs that produce various protein isoforms [36]. We were particularly interested in these high transcript complexity loci as we hypothesize they are indicative of post-transcriptional regulatory mechanisms analogous to SERPINA1 (Fig 1A). In order to analyze the transcript complexity of the COPD list and our other gene lists, we corrected for the length bias inherent in GWAS studies as well as the number of genes in each list (S2 Fig). To do so, we generated control gene sets with similar length distributions for each of the disease lists by randomly selecting length-matched genes from a set of annotated human genes [37]. Then, we tested whether these loci-length controlled measurements of transcript complexity would reveal a difference in COPD-associated gene lists versus randomized control lists.

**Fig 2:**
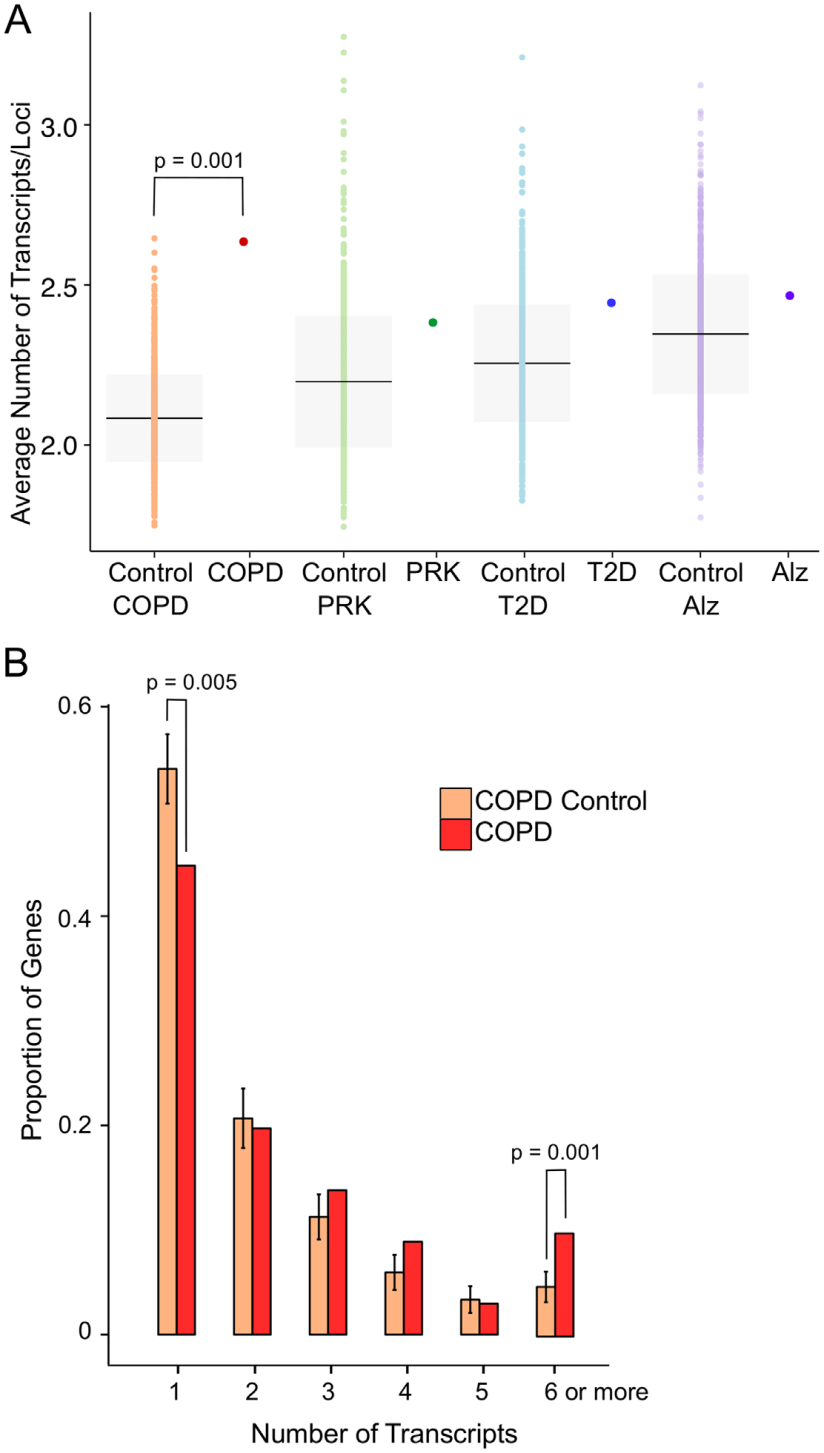
COPD-associated genes are transcriptionally complex. *(A)* We calculated the average number of transcripts per loci for length-normalized control loci and each disease-associated gene list. The control sets used as comparisons are labeled. The mean and standard deviation of the control lists are shown (black bar, grey box). Only the COPD-associated gene list is significantly different from the normalized control (p = 0.001). *(B)* We analyzed the number of gene loci that produce 1, 2, 3, 4, 5, and 6 or more transcripts and plotted these gene loci by their proportion in each list. The COPD-associated gene set has significantly fewer loci with 1 transcript and significantly more loci with more than 5 transcripts (p = 0.005 and p = 0.001).

When we performed transcript complexity analysis on our COPD gene list, we discovered that these loci produce significantly more (2.65 transcripts per gene, p = 0.001) than the expected number of transcripts from each locus (Fig 2A). This is unique to COPD-associated genes, as no significant enrichment is observed in comparable PRK, T2D, and ALZ gene lists, although these genes do tend to have higher than average transcript complexity than randomized control lists. To better characterize the bias in COPD-associated loci we categorized the loci by how many transcripts they produced. We found that COPD-associated genes have significantly fewer loci with only one transcript and significantly more loci with greater than 5 transcripts (Fig 2B, **p = 0.005 and 0.001**). The transcript complexity observed in SERPINA1 (Fig 1A) appears to be a feature of many COPD-associated genes, suggesting that alternative splicing may be an important component of COPD predisposition.

**Fig 3:**
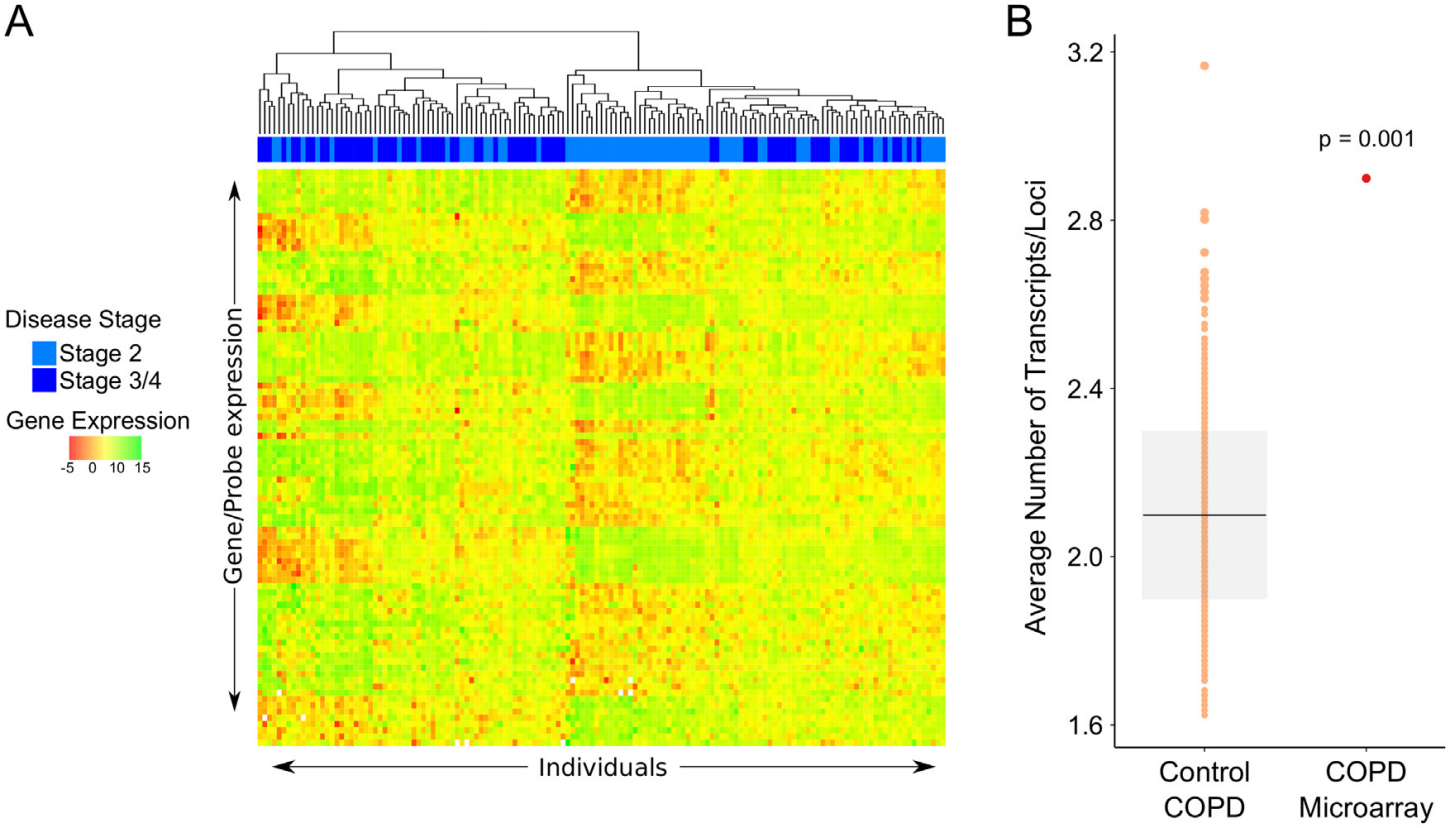
The transcript complexity enrichment of COPD-associated genes is robust. *(A)* Microarray data from the sputum of ex-smokers diagnosed with COPD stage 2, 3, or 4 identified 85 COPD-associated genes with significant differential expression (gds4265). These 85 genes are shown on the y-axis and individuals are shown on the x-axis with their COPD status marked by disease stage along the top. *(B)* We calculated the average number of transcripts per loci for these 85 differentially expressed COPD-associated genes along with length-normalized control gene sets. The mean and standard deviation of the control lists are shown (black bar, grey box). These expressed genes are significantly more transcriptionally complex than controls (p = 0.001).

Since our list of COPD-associated genes includes those genes with varying degrees of experimental support, we decided to use patient data to solidify our confidence in our gene list and re-test our hypothesis. As part of the Evaluation of COPD Longitudinally to Identify Predictive Surrogate Endpoints (ECLIPSE) Study sputum samples from ex-smokers with varying degrees of COPD (level 2, 3 or 4) were collected to compare patient gene expression via microarray with additional independent sputum samples used for PCR confirmation (gds4265) [38]. We identified 85 genes from our original list that are differentially expressed between mild, moderate and severe COPD patients (Fig 3A, Methods). We confirmed that these differentially expressed COPD genes were more transcriptionally complex than control gene sets (p = 0.001) (Fig 3B). Examples of transcriptionally complex genes, their differential expression values and their connection to COPD are listed in Table 1.

**Table 1:** Transcriptionally complex loci associated with COPD from genes expressed in stage 2 versus stages 3 and 4 COPD patients [38].

HGNC Name	Full Name	RefSeq Isoforms	Stage 2 vs 3 differential expression	Stage2 vs 4 differential expression	adj.P.Val	COPD References	Gene Function
CSF3	Colony-stimulating factor 3	5	1.16	1.02	2.62E-03	[39]	[40], [41], [42]
FAM13A	Family with sequence similarity 13, member A	5	0.35	-0.31	3.70E-03	[43], [44], [45], [46], [47], [48], [49]	[50], [51]
PDE4D	3',5'-cyclic-AMP phosphodiesterase activity and degrades cAMP	9	0.48	0.56	1.00E-04	[52], [53], [54], [55]	[56], [57]
AGER	Advanced glycosylation end product-specific receptor	10	0.49	-0.16	3.80E-02	[58], [59], [60]	[61], [62]
SERPINA1	Serpin peptidase inhibitor, clade A [alpha-1 antiproteinase, antitrypsin], member 1	11	-0.06	-0.15	1.25E-02	[55], [63], [64]	[65], [66]
TP53	Tumor protein p53	15	-0.73	-1.04	3.83E-07	[67], [68], [69], [70]	[71], [72], [73]
FUT8*	Fucosyltransferase 8 [alpha [1,6] fucosyltransferase]	5	-0.41	-0.51	6.52E-03	[74]	[75], [76]
NR3C1*	Nuclear receptor subfamily 3, group C, member 1 [glucocorticoid receptor]	15	0.51	0.27	2.00E-03		[77], [78], [79], [80], [81]
VEGFA*	Vascular endothelial growth factor A	19	0.34	0.42	6.49E-05	[82]	[83]

* Genes not included in the strongly supported COPD gene list

We removed genes that had weaker experimental support to check a subset of 151 COPD-associated genes. These strongly associated COPD genes were significantly more transcriptionally complex than control gene lists (p = 0.012) (S3 Fig). Finally, we determined how much of the transcriptional complexity within each list was due to a few genes by systematically removing the most complex genes from the full COPD list and the top three normalized control lists. We found that the original COPD list remained significantly more enriched for transcriptionally complex genes even with the top three complex genes removed (p = 0.002 to p = 0.017) (S3 Fig). This was not true of the control lists—with the top three complex genes removed none of these lists remained significantly transcriptionally complex (p = 0.231, 0.781 and 0.093) (S3 Fig). These experiments support our hypothesis that COPD-associated genes are transcriptionally complex.

### Transcriptional complexity in cystic fibrosis modulatory genes and idiopathic pulmonary fibrosis-associated genes

We chose T2D, ALZ and PRK to compare with COPD transcript complexity since we were able to curate similar sized gene lists for these diseases. Nonetheless, these diseases have very different pathologically affected tissues, so we additionally analyzed gene lists associated with the lung diseases idiopathic pulmonary fibrosis (IPF) and cystic fibrosis (CF). COPD is characterized by progressive airflow limitation while IPF is a progressive fibrosis of the lung that restricts normal breathing [84]. In comparison, cystic fibrosis is caused by mutations in CFTR, resulting in abnormal mucus and impaired lung function. We found 45 genes associated with IPF [18, 85–87] (S9 Table) and 80 genes that modulate CF [18, 88–90] Both IPF associated genes and CF modulators separately share ~31% different COPD-associated genes (S9 and S10 Tables). IPF-associated genes may be more transcriptionally complex since 20% have 6 or more transcripts, while only 8.25% of CF genes have 6 or more transcripts (S8 Fig). Likewise, the average number of transcripts per gene is 2.65 for COPD-associated genes, 2.04 for CF-associated modulators, and 2.75 for IPF-associated genes. Due to the small number of genes currently associated with IPF and CF we cannot statistically establish the role of transcriptional complexity in these diseases. However, the high levels of transcriptional complexity in COPD and IPF suggest that post-transcriptional processes in the lung should be further investigated as potential mediators of disease.

### COPD-associated transcript complexity is not generated from non-canonical splicing, exon size or transcript size

The high transcript complexity observed in COPD-associated genes suggests that alternative splicing is an important aspect of the post-transcriptional regulatory program of COPD-associated genes. Therefore, we further scrutinized our COPD gene list to identify transcript features that would lead to alternative splicing. First, we analyzed the percentage of strict canonical splice sites within the donor and acceptor regions of control gene sets. A difference in the percentage of canonical splice sites within COPD-associated genes would suggest that the observed transcript complexity is generated through non-canonical splicing. However, we discovered that COPD-associated gene splice sites are not significantly different from control splice sites (S4 Fig). We also found that there are no significant differences between COPD-associated genes and the length of the mature mRNAs or the number of large exons within the genes (S5 and S6 Figs). Thus, the high levels of alternative splicing in these genes must arise from other, unidentified, characteristics.

### COPD-associated gene loci have unusual GC content and splicing patterns

**Fig 4:**
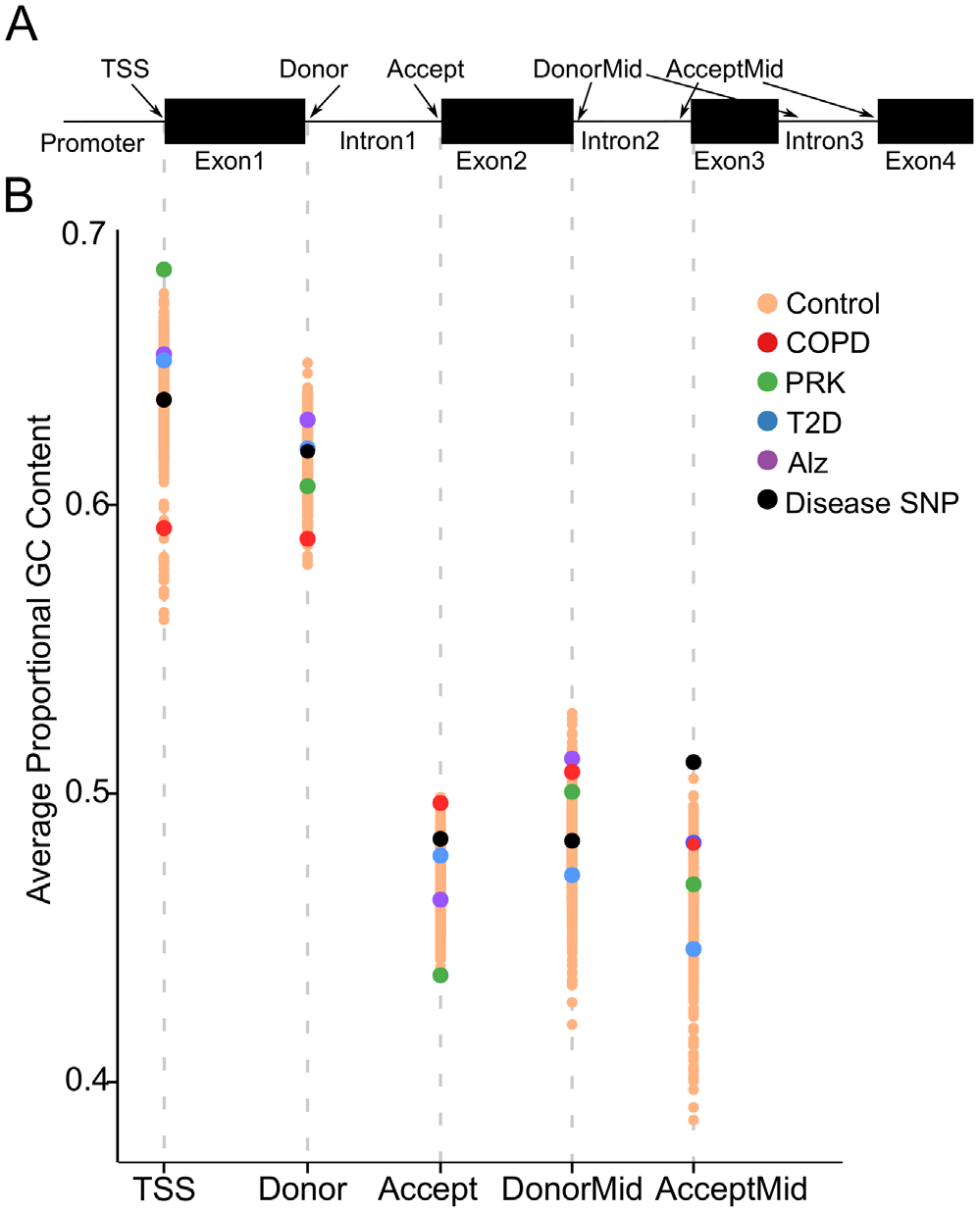
COPD splice junctions have lower GC content than expected. *(A)* Diagram of a representative gene with the regions of interest labeled, including the transcription start site (TSS), the first donor splice site (Donor), the first acceptor splice site (Accept) and the subsequent internal donor and acceptor splice sites (DonorMid and AcceptMid). *(B)* We calculated the average GC content for the 60 nucleotides surrounding each region in COPD-associated genes as well as genes associated with other diseases (PRK, T2D and ALZ). As a comparison, we also analyzed the GC content of genes with disease-associated SNPs within the TSS or splice junctions (black dots).

Next, we explored the GC content around the first exon and splice sites. Within a gene, the region around the transcription start site (TSS) generally has high GC content, as do the splice junctions surrounding the first intron [27, 91, 92]. We determined the GC content within the sixty nucleotides surrounding the TSS, the first 5’ donor splice site (Donor), the first 3’ acceptor splice site (Accept) and the remaining splice acceptor and donor populations (DonorMid and AcceptMid) (Fig 4A). While COPD-associated genes are not significantly different than control genes at each individual boundary, they trend toward less abrupt differences between splice regions. To quantify this we analyzed the change in GC content surrounding the first intron (the difference between the first 5’ donor splice site and the first 3’ acceptor splice site). In COPD-associated loci this range narrows to 9.52%, and is significantly smaller than control samples (17.1%, p = 0.004). Unlike COPD gene loci, the other gene sets we analyzed have a broader range of GC content (Fig 4B, Table 2). In addition, known SNPs associated with disease that fall within splice junctions had a normal Donor-Acceptor breadth (15.2%) (Table 2). As differences in GC distribution are linked to gene evolutionary age and tissue-specific expression, we propose that this characteristic sets apart a group of complex genes with important roles in COPD etiology [93].

**Table 2:** In COPD the difference between the average GC content of the transcription start site and the first acceptor splice site is significantly smaller than expected (p = 0.004).

List	Average Difference	Standard Deviation
Control	17.1	1.76
COPD	9.52	-
Parkinson's Disease	24.4	-
Type 2 Diabetes	17.1	-
Alzheimer's Disease	18.8	-
Disease associated SNPs	15.2	-

**Fig 5:**
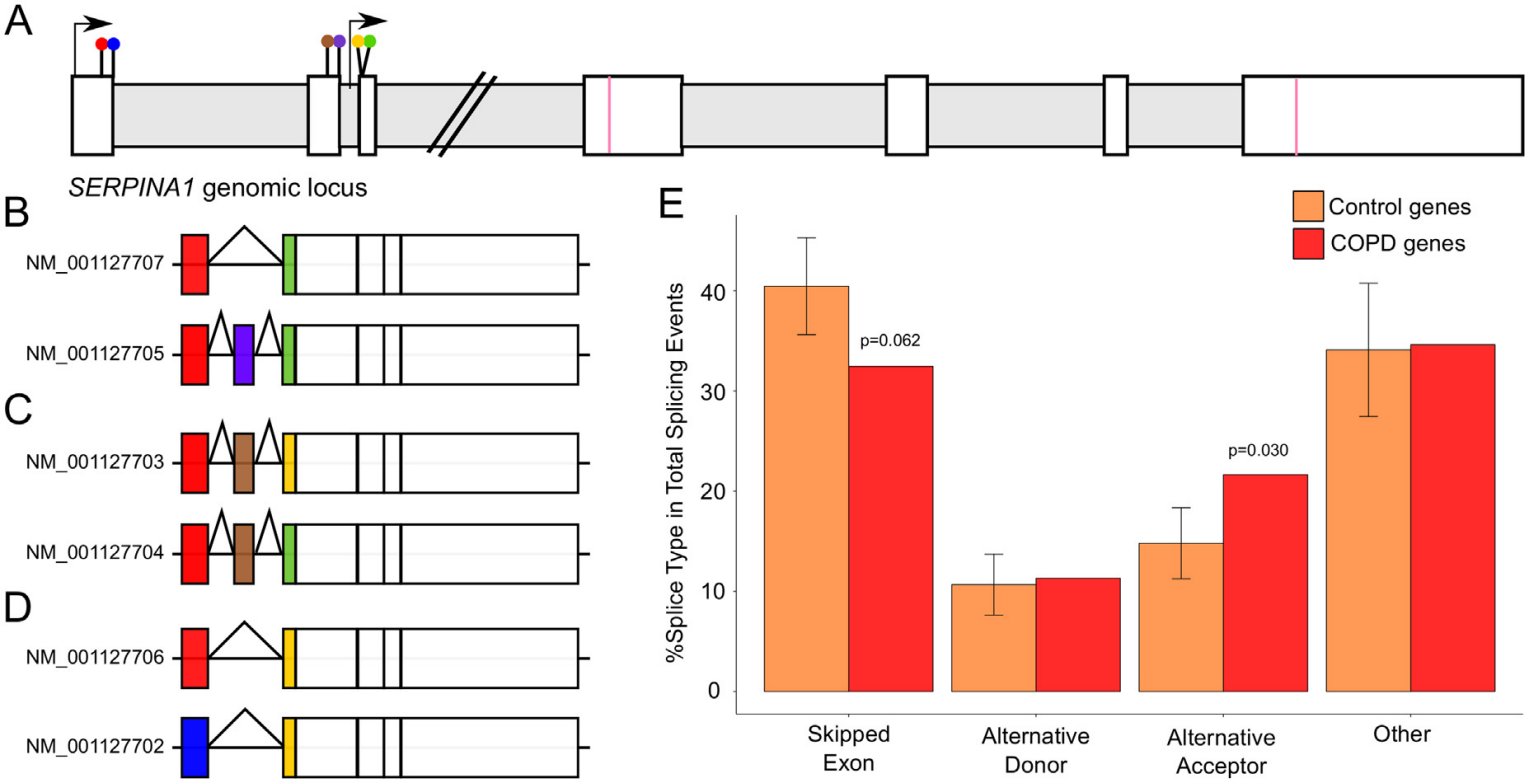
Skipped exons and alternative acceptor splicing events are differentially enriched in COPD genes. *(A) SERPINA1* contains a number of different splicing events including *(B)* skipped exons, *(C)* alternative donors and *(D)* alternative acceptors. *(E)* On average, COPD genes contain fewer skipped exons and significantly more alternative acceptor splice events (p = 0.030) per total splice events in comparison with normalized reference genes.

Our observations raise the question as to whether COPD-associated genes produce transcripts with atypical splicing patterns. Alternative splicing in higher eukaryotes is characterized primarily by exon skipping, but also includes alternative acceptor and donor splice sites and a variety of other complex events incorporating these and other types of splicing [94]. The *SERPINA1* gene is characterized by a very large number of potential splice sites (Fig 5A). For example, *SERPINA1* transcripts contain exon skipping events (Fig 5B) as well as alternative donor (Fig 5C) and alternative acceptor sites (Fig 5D). We analyzed control sets of genes to determine what a typical splicing pattern is like for gene loci. On average, we ascertained that alternatively spliced genes normally have around 41% skipped exons, 11% alternative donor sites, 15% alternative acceptor sites and 34% more complex events (Fig 5E). In comparison, alternative transcripts produced from the COPD-associated genes have fewer skipped exons (28%) and more alternative acceptor sites (22%), such as the event depicted in *SERPINA1* transcript (Fig 5D).

### COPD-associated splicing isoforms are expressed in relevant tissues in a regulated fashion

**Fig 6:**
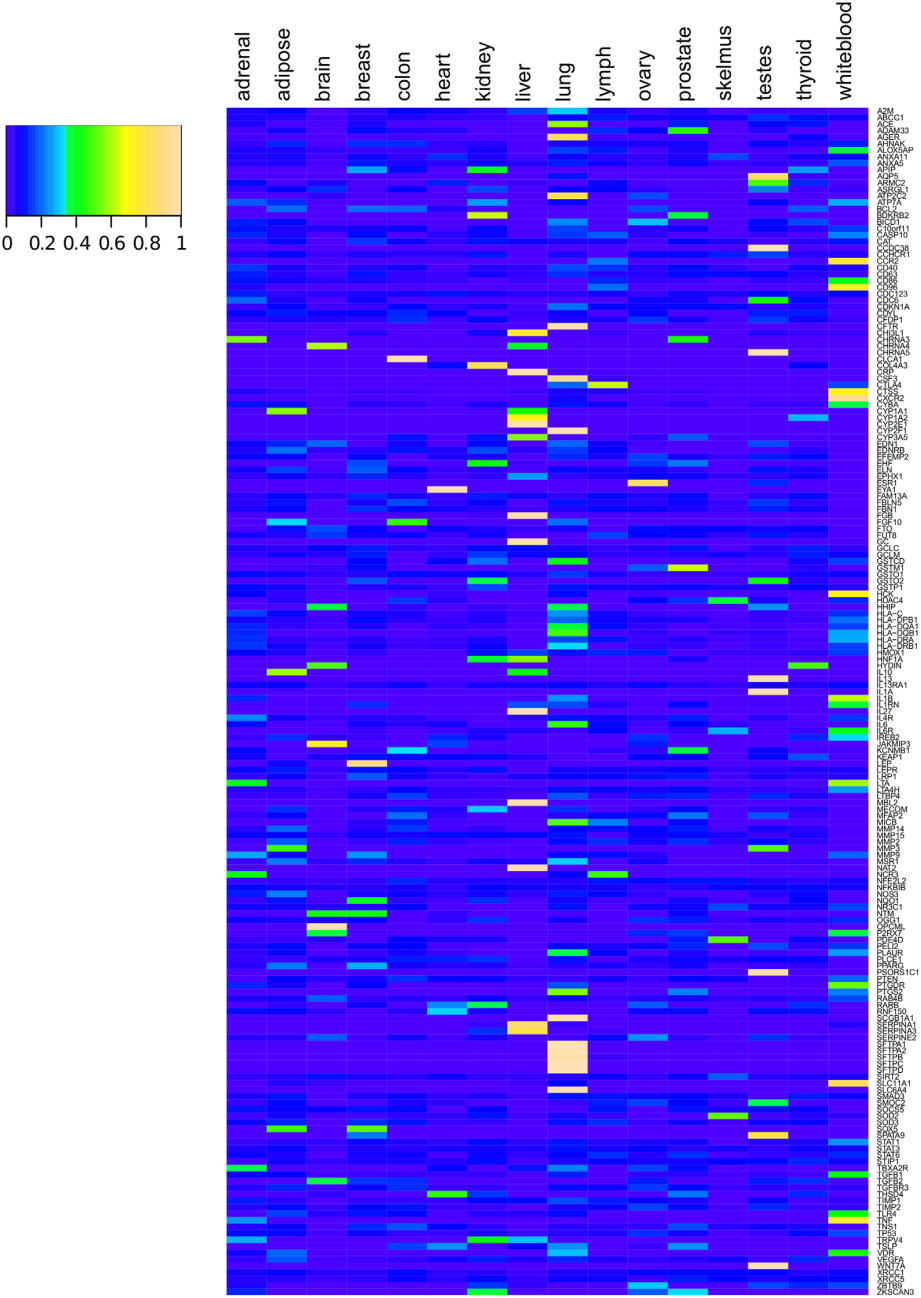
Highly expressed COPD-associated gene transcripts are enriched in lung tissue. The highest expressed transcript from each COPD-associated gene is shown as a percentage of expression in each of the 16 tissues from BodyMap. COPD-associated gene expression is highest in lung, white blood cells, testes and liver (14.5%, 9.9%, 9.0% and 8.7%), respectively. Other COPD-associated transcripts not in these tissues are still tissue specific and may be detrimental if expressed in the lung. Splice variants were determined as alternative splice sites through ASprofile [95].

Although many genes have documented alternative transcripts, we do not currently have a complete picture of when or where these isoforms are expressed. We have extensively demonstrated that COPD-associated genes are documented to have an unusually high number of transcripts per loci, but we wanted to know whether these transcripts were used in the lung or liver tissue (where *SERPINA1* is expressed and secreted) [65]. We analyzed the alternative splicing profiles generated from the Illumina BodyMap dataset. Looking at the highest expressed splice variant from each loci we saw that each was generally dominant in one or two tissues, rather than broadly expressed across the whole human body (Fig 6, S1 Table: **BodyMap Tissues for a list of genes and tissue distribution**). In addition, expression of the highest expressed variant was frequently found in lung tissue (14.5%), as expected for genes with a role in COPD. This enrichment of COPD-associated highly expressed variants is significant (p < 0.001) as the average enrichment of highly expressed variants in lung tissue from control gene lists is 4.82% (S9 Fig). The next enriched tissues in COPD associated-gene transcripts were white blood cells, testes and liver tissue (9.9%, 9.0% and 8.7% respectively). The tissue where the most prevalent transcript isoform is expressed is listed for each gene in S1 Table. Our results support the relevance of our COPD list to the disease.

**Fig 7:**
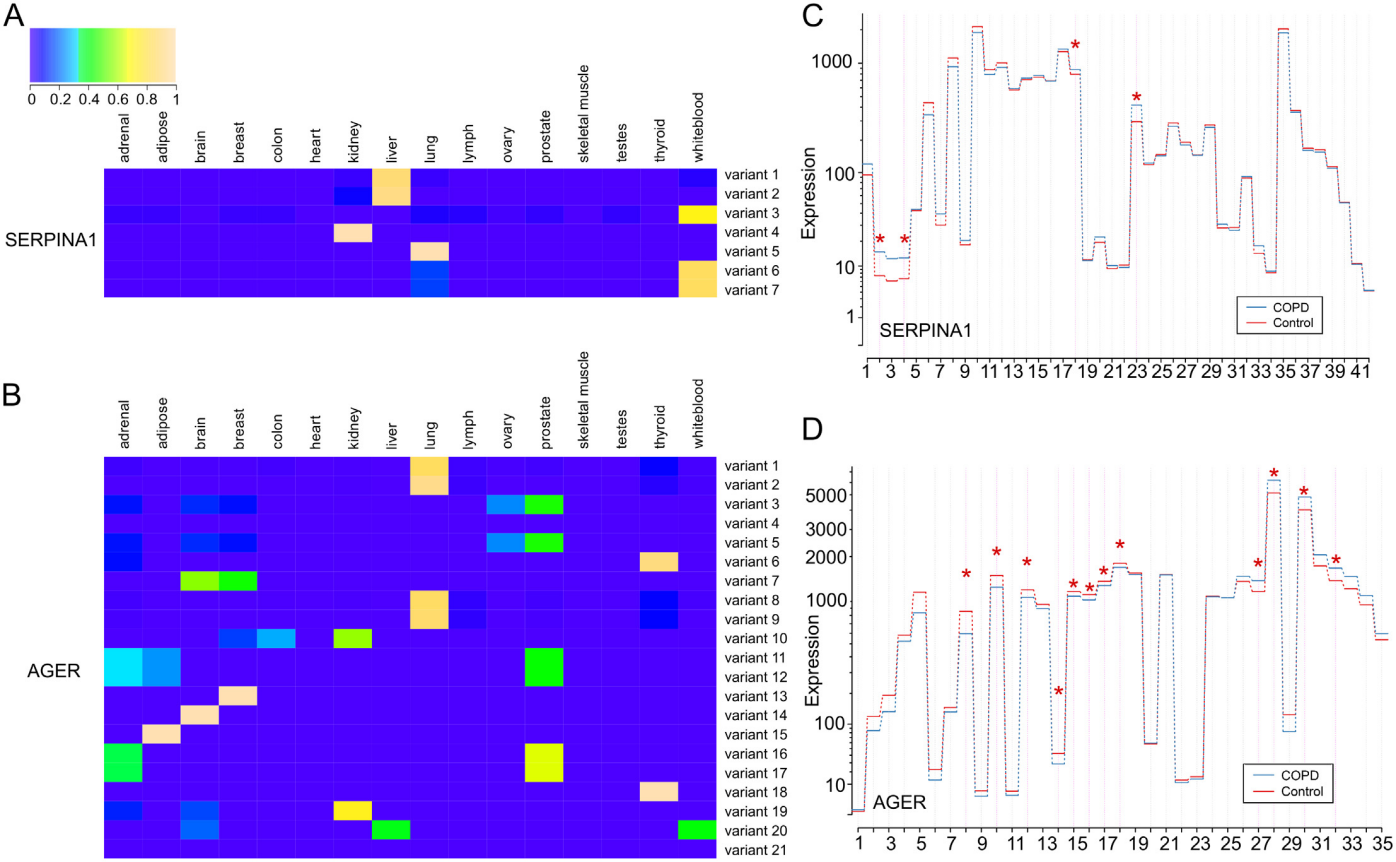
Usage of *SERPINA1* and *AGER* splice variants by tissue and COPD status. *(A)* The expression of each splice variant in *SERPINA1* was normalized across tissues. The pattern of expression shows that these splice variants are not broadly used in every tissue, but specific to the kidney, liver, lung and white blood cells. *(B)* Likewise, *AGER* is expressed in nearly every tissue tested, but splice variants are have specific patterns that imply regulation. Splice variants were determined as alternative splice sites through ASprofile [95]. *(C)* RNA-seq data from control subjects and COPD patients indicate that in *SERPINA1* four exons are significantly differentially used (p < 0.05). *(D)* In the *AGER* gene twelve exons are differentially expressed in COPD patients compared to normal controls (p < 0.05). Exon usage was generated with DEXSeq [101].

In addition to examining general characteristics of COPD-associated gene expression, we wanted to analyze several genes in depth to inspect all of their splicing variants. To do so, we chose to scrutinize our archetypical COPD gene–*SERPINA1*. The highest expression of *SERPINA1* occurs in the liver, and liver expression is mainly confined to two splice variations. However, in other tissues these splice variants are not used and alternative splicing occurs (Fig 7A). We then examined *advanced glycosylation end product-specific receptor* (*AGER*). We chose this gene because of the evidence underlying its association with COPD, as well as its plethora of splice variants. The protein product of *AGER* is a multiligand cell surface receptor with links to many diseases involved with inflammation, including COPD [96–99] (Table 1). In addition, *AGER* is well documented to produce many alternative splice variants [100]. Similar to *SERPINA1*, we saw that *AGER* splice variants are highly regulated between tissues (Fig 7B). Several splice variations are present in COPD relevant tissues, like the lung, white blood cells and liver.

Furthermore, we decided to determine if alternatively spliced transcripts are differentially expressed in COPD patient lung tissue. Using RNA-seq data from the lung tissue of 26 COPD subjects and 26 controls with normal spirometry, a subset of data generated by Kim et al [102], we analyzed differential expression of highly complex, COPD-associated genes. We tested each gene in our COPD gene list with DEXSeq for differential exon usage (DEU), which measures the change in the relative exon usage between sample sets [101]. The transcript complexity contributing to DEU represents alternative TSS and polyadenylation sites as well as alternative splicing. For each gene, we report the total number of exons and the number of these exons that are differentially expressed in COPD and control subjects (FDR < 0.05) (S1 Table). Many COPD-associated genes, such as AGER and SERPINA1, exhibit significant DEU in multiple exons (Fig 7C and 7D), supporting our conclusions that transcriptional complexity is a feature of COPD etiology. Previously, Kim et al., identified specific alternative splicing patterns that were prevalent in COPD subjects compared to control subjects. Our findings are consistent with this finding, which further supports the hypothesis that transcript complexity is an important aspect of COPD [102].

## Discussion

Current databases have been refining gene annotations for decades and, more recently, incorporating a flood of RNA sequencing data. One aspect arising from this work is a plethora of transcripts produced from alternative splicing. We provide evidence that COPD-associated genes are enriched for highly complex mRNAs, implying that the development of COPD might be influenced by malfunctions in alternative splicing. This transcriptional complexity is evident in two commonly used transcript reference databases, NCBI RefSeq and Ensembl (S10 Fig). We expected that other chronic diseases might also be enriched for transcriptionally complex genes. However, we did not find any significant enrichment in our disease gene sets for Type 2 Diabetes, Parkinson’s or Alzheimer’s disease even though they all trended higher than the average of their control sets. We did find that idiopathic pulmonary fibrosis (which shares more than 30% of the same genes as COPD) trends toward increased transcript complexity, while cystic fibrosis (which also shares more than 30% of the same genes as COPD) does not (S9 and S10 Tables). However, these data sets are small and will require further investigation.

Interestingly, an analysis of all genes with six or more transcripts indicates that transcriptionally complex genes are generally more enriched in disease databases, like the NHGRI catalog and SNP4Disease, than genes with less than six transcripts (S7 Fig). These databases collect information from a wide variety of diseases, including cancers and chronic diseases. The enrichment of highly transcriptionally complex genes in these disease-focused databases could reflect the susceptibility of these genes to mis-regulation that can lead to disease.

COPD-associated genes were significantly enriched with transcriptionally complex genes. COPD is a long-term degenerative disease with a strong environmental component; specifically damage caused by cigarette smoking. We do not fully understand how COPD develops, but it is clear that the immune system plays an important role. For example, T cells are found at higher numbers in the lungs of COPD patients and damaged epithelial cells secrete a variety of inflammatory signals [103]. The human adaptive immune system has been developing since the early vertebrates, but is still a relatively new and complex biological system, with significant dependence on alternative splicing for fine-tuning response [104–106]. It is possible that although transcriptional complexity allows for greater flexibility and control in complex systems, it is more likely misregulated, particularly in systems that depend heavily on alternative splicing. However, a deeper understanding of the biological causes of COPD, as well as further research into the function and regulation of transcript variants, will be required to understand why COPD is associated with more transcriptionally complex genes.

The mechanisms that control alternative splicing are still an active area of investigation. We explored several genetic aspects of COPD-associated genes to narrow down the features that can affect a gene’s ability to produce multiple isoforms from the same or similar transcripts. Unexpectedly, we found that COPD-associated genes trend toward constant GC-rich regions even though alternative splicing is generally correlated with high GC content around splice junctions. We also inspected transcript size, number of large exons and canonical/noncanonical splice sites, but did not find any differences between COPD-associated genes and control gene sets. It is possible that COPD genes may rely on enhancer elements or other regions outside of splice junction to control splicing activity. When we analyzed the types of splicing taking place within COPD-associated transcripts we discovered an increase in alternative acceptor sites. However, what drives splicing in these genes is not yet clear.

We used microarray expression data to detect broad gene expression changes during COPD progression as a way to confirm the importance of a subset of genes in COPD. However, since most microarray platforms do not detect splice isoforms, we also used the Illumina BodyMap RNA-seq alternative splicing profile data set to explore tissue specific expression of mRNAs. As expected, COPD-associated genes are highly expressed in lung tissue. More importantly, we saw tissue specific isoform expression—supporting the notion that splice variant expression is regulated and that transcripts have different biological roles. We also looked at alternative exon usage from a recent RNA-seq experiment with COPD and control patients [102]. When we looked at transcripts from COPD-associated genes in these people, we found that many of the exons were significantly differentially expressed in COPD patients in comparison to people with normal lung function.

COPD is both prevalent and incurable, but studies to identify the molecular causes of COPD lag behind other diseases like diabetes or cancer. Based on our findings that COPD-associated genes have a high level of transcript complexity, we propose that alternative splicing may be of particular interest in the study of COPD etiology.

## Materials and Methods

### Disease List Creation

We searched NCBI PubMed for reviews with the terms ‘genetics’ and each disease. We progressed from the most recent review article to the next, compiling a list of genes. After three reviews minimal unique genes were added to the gene lists for COPD, Parkinson’s disease and Type 2 Diabetes. To identify Alzheimer’s-associated genes we used four recent reviews, as a significant number of new disease-associated genes were added with another publication. The identified reviews spanned 2009 to 2014. Gene candidates were updated with the HUGO Gene Nomenclature Committee (HGNC) nomenclature and combined into literature review lists. In addition, we used the National Human Genome Research Institute (NHGRI) catalog of published GWAS [18]. We queried the catalog using supplied disease/trait terms, such as ‘Parkinson’s disease’ and derivatives, such as ‘Parkinson’s disease (age of onset)’. We updated these genes with recent HGNC nomenclature and combined them with each literature review list.

### Creation of Normalized Control Lists

To generate control gene sets normalized by length, we obtained genomic coordinates from the UCSC Genome Browser (hg19) [107, 108]. Length for each loci was determined as the maximum span encompassing all transcripts of the gene. Misannotated and pseudogene loci with excessively large sizes were eliminated by removing loci with more than 1 million base-pairs. We then randomly selected genes from the UniProt-GOA reference human genes from the GO Consortium's Reference Genome initiative [37] so as to obtain a comparable length distribution (within 10% variance) to the disease test with the same number of genes. This operation was repeated 1000 times to establish statistics on how often the disease set was more transcriptionally complex than the random gene list. The reference gene set used is composed of over 17,000 gene loci, allowing us to establish independent gene lists for statistical evaluation. A small subset of disease genes had very small loci (e.g. miR499) and were not considered in the analysis.

### Gene to Transcript Calculations

The number of transcripts from a locus was calculated by counting the number of unique UCSC Genome browser (hg19) RefSeq transcript IDs linked to the HGNC gene name [107, 108]. Alternatively, we used Ensembl transcript IDs, available from BioMart, linked to HGNC names to develop a pool of control genes. To calculate the statistical significance of the controls versus the disease-associated lists we performed boot-strapping with 1000 different randomized control lists for each disease. We used this data to calculate the number of genes producing 1, 2, 3, 4, and 5 or more transcripts.

### Differential Gene Expression Analyses

Microarray data analyzing gene expression changes in COPD patients are available from the Gene Expression Omnibus (GEO) NCBI browser. We chose to analyze a large dataset (148 samples) with gene expression information collected from the sputum of ex-smokers diagnosed with COPD stage 2, 3 or 4 (gds4265) [38]. To analyze the data we used R bioconductor with Biobase, GEOquery to download and modify to microarray to useable form and empirical Bayes methods (limma) to determine which genes were differentially expressed by disease state [109–111]. We compared genes with significant expression (adjusted p-value greater than 0.05) to genes associated with COPD and tested these 85 genes for transcript complexity. The p-value adjustment performed by Singh, et al. corrected for age, gender and batch of patients [38].

### GC content analyses

To obtain sequence information around splice junctions, we downloaded exonic sequences with additional 30 nucleotides upstream and downstream from the UCSC database (hg19) [107, 108]. The first 60 nucleotides of the first exon were used to calculate the percentage of G and Cs around the transcription start site (TSS). The last 60 nucleotides of the first exon were the first donor site (Donor) and the first 60 nucleotides of the second exon were the first acceptor site (Accept). All the remaining 5’ junctions were combined as the DonorMid calculation. The AcceptMid value was the combination of all the 3’ junctions with the exception of the first and last exons. Control calculations were based on non-normalized lists of 206 gene loci from the UniProt-GOA reference human gene set [37]. To calculate the statistical significance of the controls versus COPD we performed boot-strapping with 1000 different randomized control lists.

### Splice type analyses

We employed the AStalavista program to analyze splicing patterns [112]. The UCSC Genome browser (hg19) was used to obtain gtf files for the COPD-associated genes as well as control length-normalized genes in each of 1000 lists [107, 108]. These genes were analyzed for all splicing events with AStalavista and the number of skipped exon; alternative donor, alternative acceptor and other splice types were extracted from this information. The gene UTY was removed from the control set because of its abnormally large number of splice events. Splice type values were normalized by the total number of splicing events. We analyzed 1000 different randomized control lists to bootstrap the significant between the control and COPD genes.

### BodyMap Tissue Specific Expression of mRNA Isoforms

We used the Alternative Splicing profiles developed with the Illumina BodyMap RNA-seq dataset to investigate how COPD-associated gene transcripts are expressed across tissues. The BodyMap data was prepared from 16 different human tissues, subjected to polyA purification, fragmented and random primed before 2x50 sequencing on HiSeq2000 with 1 lane for each tissue (raw data available from ArrayExpress, E-MTAB-513). The approximately 80 million reads for each tissue were aligned to the human genome, hg19, and turned into fragments with Cufflinks [95] (alternative splicing profiles available from http://ccb.jhu.edu/software/ASprofile/data/). ASprofile uses pairwise comparison of splicing events to define splice sites within each gene based on Ensembl annotations and sequencing data. Additional programs contained in the ASprofile suite calculate normalized expression for each tissue in a comparable fashion. We used ASprofile to map the percent expression in each tissue of the highest expressed transcript from each of the COPD-associated genes. We also investigated *SERPINA1* and *AGER* and mapped the percent expression in each tissue for all of the documented splice variants in these genes. To calculate the statistical significance of COPD lung enrichment we performed boot-strapping with 1000 different randomized control lists, selected the ASprofiles for the genes in each list, and computed the number of highest expressed variants expressed in the lung from each list. We used the same protocol to compute T2D, ALZ and PRK lung enrichment.

### Differential Exon Usage in COPD Patients versus Control Subjects

We used publically available data generated as part of a study of COPD patients in comparison to subjects with normal spirometry [102]. The samples were prepared from lung tissue from 98 COPD patients and 91 controls, subjected to polyA-selected RNA extraction, fragmented, and primed with random hexamers before 2x50 sequencing on HiSeq2000 (raw data available from NCBI Gene Expression Omnibus (GEO) through accession number GSE57148). Read quality and alignment were verified with FastQC and Picard. They were aligned to the human genome, hg19, and expression was measured with Cufflinks 2.0.0. A subset of 26 control samples and 26 COPD-affected samples were randomly selected for analysis of differential exon usage (DEU) in our gene list using DEXSeq [101]. For each gene, a list of all transcripts were flattened into exon "counting bins", which are whole exons or fragments of exons that have differing boundaries between transcripts. For each exon bin, the number of reads that map to it were counted; if the read overlapped several exons, it was counted in multiple bins. The relative exon usage was calculated as a proportion of the number of transcripts from the gene that contain the exon per the number of all transcripts from the gene. Using read coverage for each bin, dispersion values were calculated to then test for significant DEU between COPD and control. To correct for the multiple hypothesis testing, the p-value was adjusted by the Benjamini-Hochberg algorithm and reported as a false discovery rate.

### GLAD4U and SNP4Disease COPD List Creation

We accessed the SNP4Disease database of disease-associated SNPs (http://snp4disease.mpi-bn.mpg.de/) as an additional source of disease information. To develop the comprehensive database list of genes, we queried the catalog using all supplied disease terms, such as ‘Respiratory Tract Diseases’ and ‘Virus Diseases’. For the COPD-associated genes, we searched the database for ‘Pulmonary Disease, Chronic Obstructive’. We used the gene symbol associated with each SNP. To explore text-mining results, we used Glad4U (http://bioinfo.vanderbilt.edu/glad4u/) [20]. We searched PubMed through this application for the term ‘Chronic Obstructive Pulmonary Disease’. We updated all gene lists with the most recent HGNC nomenclature before further analysis.

### Removal of Weakly Correlated COPD Genes

We took advantage of a comprehensive literature review to sort COPD-associated genes by evidence [15]. We removed all genes with more negative than positively correlating studies and all genes with equal numbers of negatively and positively correlating studies. This resulted in a subset of 151 remaining COPD-associated genes, and eliminated genes with negative or conflicting evidence. We did not attempt to remove genes based on journal of publication or study type.

### Transcript and Exon Length Comparisons

Genomic coordinates from UCSC detailing the position of all exons within a gene for control and disease gene lists were used to count how many exons larger than 200 base-pairs were in each transcript [107, 108]. We compared the average number of large exons between the COPD-associated transcripts and control transcripts using boot-strapping with 1000 control lists. In addition, we used these coordinates to analyze the total length of transcripts in control and disease lists.

### Canonical vs. Non-canonical Splice Site Analysis

We downloaded exonic sequences with additional 30 nucleotides upstream and downstream from UCSC database. We selected the 60 nucleotides 5’ and 3’ of each exon and looked for the presence of a strict splice site within this region. The 5’ region of the first exon and 3’ region of the last exon were excluded. For the 5’ site we used the sequence GGTAA / GGTGA. For the 3’ site we used the sequence CAGGT / TAGGT. Control calculations are based on non-normalized lists of 206 gene loci from the UniProt-GOA reference human gene set using boot-strapping with 1000 random lists.

### Analysis of Disease Enrichment in Transcriptionally Diverse Genes

We separated a set of 804 genes with more than 5 transcripts from the UniProt-GOA reference human gene set. Using genes with 1 to 5 transcripts to make control sets of genes, we calculated the number of disease-associated genes within 1000 control sets and within the gene set with more than 5 transcripts to boot-strap the significance of our findings. We performed these calculations for both the NHGRI GWAS catalog of disease-associated genes and the SNP4Disease database of disease-associated genes.

## Supporting Information

S1 Fig Non-curated COPD-associated gene sources contribute unique candidates. SNP4Disease results for genes associated COPD and other related disorders, resulting in a large pool of candidate genes that overlap with text-mining Glad4U results for COPD (left). The COPD-associated gene list used in the main body of this paper is not fully identified by either Glad4U or SNP4Disease results. Both SNP4Disease and Glad4U contribute additional genes that are not commonly referenced in COPD literature or the NHGRI database (right).
(TIFF)

S2 Fig GWAS studies select for longer gene loci. *(A)* We calculated the average length for equal sets of genes from the UniProt-GOA reference human gene list (orange) and the NHGRI GWAS catalog (yellow) and plotted a histogram of their length distribution. The NHGRI GWAS catalog has much larger average gene loci lengths, therefore in all subsequent analyses we controlled for length. *(B)* We calculated the average length for equal sets of genes from the UniProt-GOA reference human gene list (orange) and genes from the SNP4Disease GWAS database (yellow). Lists of gene loci selected from the SNP4Disease database are larger than gene loci selected from the reference set. *(C)* We measured the average gene loci size of reference gene lists as well as the combined disease lists (All) and literature review (L) and GWAS (G) components for all four diseases. The mean and standard deviation of the average loci size of the control lists are shown (black bar, gray box). Compared to PRK, T2D and ALZ, COPD associated loci are the shortest and fall within the expected size of control gene sets.
(TIFF)

S3 Fig The transcript complexity enrichment of COPD genes is robust. *(A)* Genes with weak or mixed connection to COPD were removed from the original list and the resulting set was significantly more transcriptionally complex than length-normalized control gene lists. *(B)* The transcript complexity in the complete COPD list remains significant even when the top transcript producing genes are removed, unlike the top three control lists treated in the same fashion. The p-values are shown in all panels.
(TIFF)

S4 Fig COPD-associated genes transcript splice sites are composed of the expected number of canonical splice sites. COPD-associated 5’ transcript splice sites contain similar percentages of canonical donor splice sites as control 5’ transcript splice sites (left). The percentage of canonical COPD-associated transcript splice sites at the acceptor site also falls within the expected percentage range of canonical sites, as determined by analyzing the 3’ acceptor splice sites from reference gene sets (right).
(TIFF)

S5 Fig Length-normalization selects control lists with similar length distributions as their template disease lists. *(A)* We measured the average gene loci size of normalized reference gene lists and the combined disease lists. The mean and standard deviation of the average loci size of the control lists are shown (black bar, grey box). *(B)* The average size of COPD-associated mRNAs falls within the range of the average mRNA size produced by length-normalized reference genes. Similar calculations were performed for Parkinson’s Disease, Type 2 Diabetes and Alzheimer’s Disease with controls normalized to each disease list. Disease-associated mRNA average size is within the expected values for all diseases tested.
(TIFF)

S6 Fig COPD-associated transcripts contain the expected number of large exons. The number of large exons (>200 bps) was calculated for each mRNA and then averaged for all mRNAs produced from each gene list. The average number of large exons per mRNA for the COPD-associated gene list falls within the range of the average number of large exons found in reference gene sets.
(TIFF)

S7 Fig Genes with more than 5 transcripts are more likely to be associated with disease. We counted the number of disease-associated genes (identified from public GWAS databases) with 1–5 transcripts compared to those with more than 5 transcripts. Using NHGRI defined disease genes (left), we found that genes with >5 transcripts were significantly enriched for disease-associated genes (p < 0.001). We discovered similar effects when we compared these genes to the SNP4Disease gene list (right), with the gene list of genes with >5 transcripts containing significantly more disease-associated genes (p < 0.001).
(TIFF)

S8 Fig IPF has a high number of genes with 6 or more transcripts. 20% of IPF-associated genes (red) and 10.68% of COPD-associated genes (green) have 6 or more transcripts, while only 8.25% of CF genes (orange) have 6 or more transcripts. The average percentage of total genes with each number of transcripts is shown for 1000 randomized control lists (blue) with the standard deviation shown as a grey ribbon. The low number of genes in the IPF and CF lists impede calculating statistical significance.
(TIF)

S9 Fig Expressed COPD transcript isoforms are enriched in lung tissue. Expression of the most prevalent isoforms of COPD-associated gene transcripts occurred in lung tissue (14.5%) while the percent of isoforms expressed in lung tissue from randomized controls as well as from ALZ, PRK and T2D was much lower (4.82, 4.32, 4.25 and 5.74% respectively). This enrichment in COPD-associated isoforms is significant (p < 0.001).
(TIF)

S10 Fig COPD-associated genes are significantly more transcriptionally complex than control datasets. *(A)* The number of transcripts per gene was calculated using Ensembl defined genes and transcripts for 1000 lists of 206 Ensembl genes as well as the COPD-associated genes. *(B)* The number of genes with 1,2,3,4,5 and 6 or more transcripts are displayed as an average for 1000 lists of control genes as well as the list of COPD-associated genes.
(TIF)

S1 Table COPD-associated genes and information. (XLS)

S2 Table SNP4Disease and Glad4U genes associated with COPD. (XLSX)

S3 Table Genes associated with Parkinson's disease. (XLSX)

S4 Table Genes associated with Type 2 Diabetes. (XLSX)

S5 Table Genes associated with Alzheimer's Disease. (XLSX)

S6 Table Overlap between Literature Reviews in disease-associated gene loci and control sets. (XLSX)

S7 Table Overlap between Literature Reviews in Alzheimer's associated gene loci and control sets. (XLSX)

S8 Table Overlap between Literature Review and GWAS identified genes in disease and control sets. (XLSX)

S9 Table Genes associated with idiopathic pulmonary fibrosis. (XLSX)

S10 Table Cystic fibrosis modulatory genes. (XLSX)

S11 Table FPKM expression of exons stratified by expression in COPD and control patients. (XLSX)
