# Supplementary figures and images for: Increased Transcript Complexity in Genes Associated with Chronic Obstructive Pulmonary Disease
Source: PLoS One. 2015 Oct 19;10(10):e0140885. doi: 10.1371/journal.pone.0140885 (PMC4610675; doi:10.1371/journal.pone.0140885)

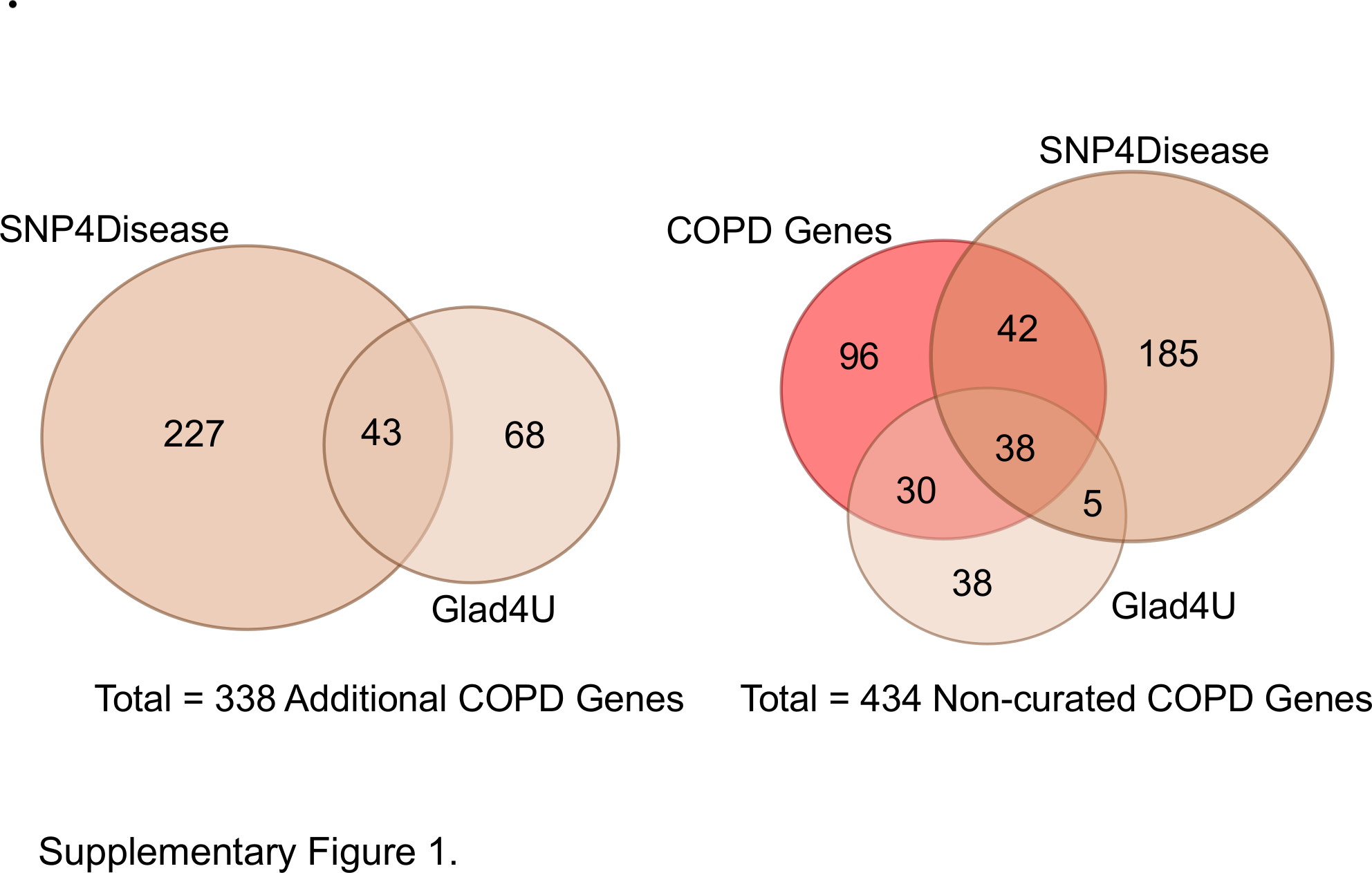

Supplement: S1 Fig — SNP4Disease results for genes associated COPD and other related disorders, resulting in a large pool of candidate genes that overlap with text-mining Glad4U results for COPD (left). The COPD-associated gene list used in the main body of this paper is not fully identified by either Glad4U or SNP4Disease results. Both SNP4Disease and Glad4U contribute additional genes that are not commonly referenced in COPD literature or the NHGRI database (right). (TIFF) [file pone.0140885.s001.tiff]

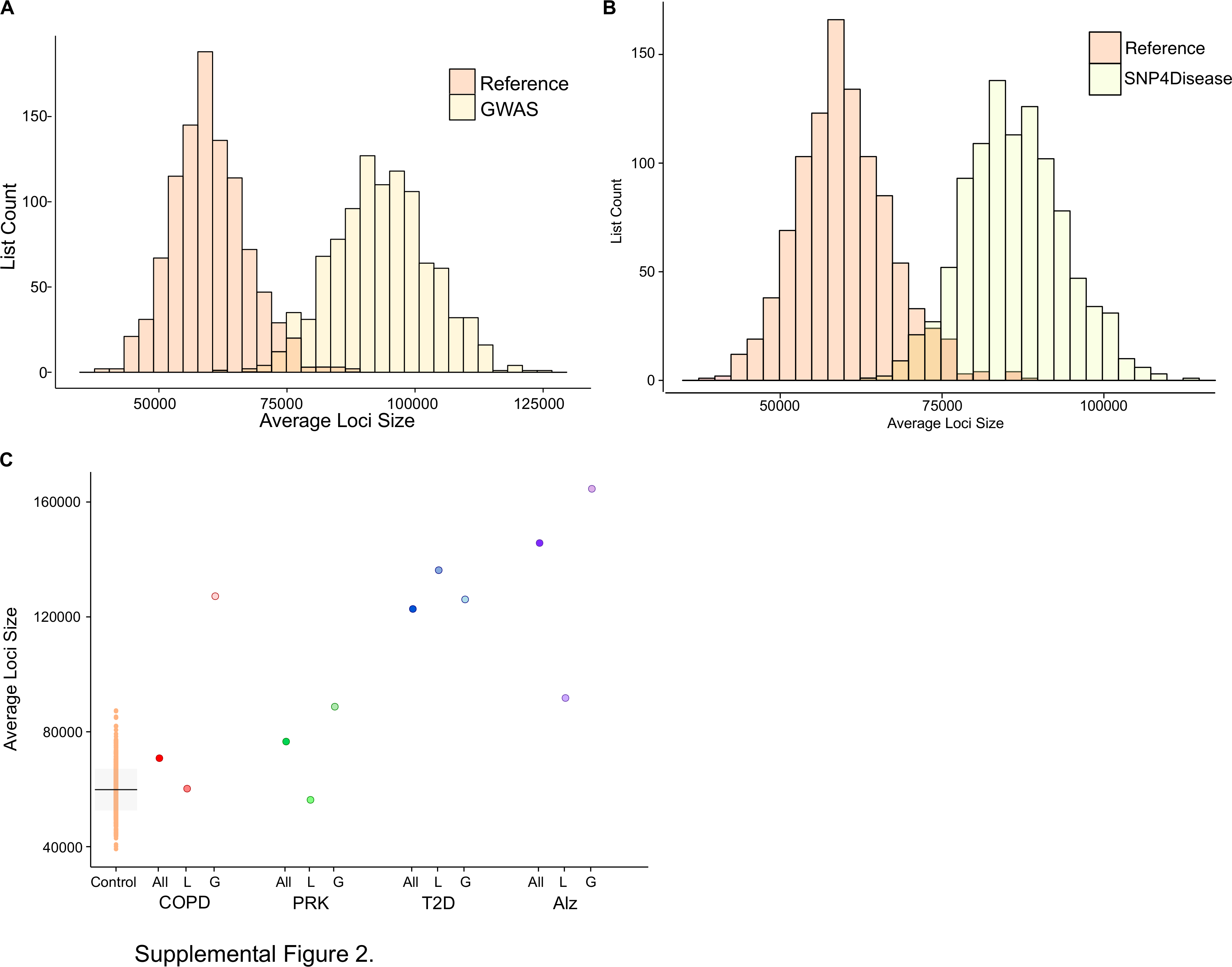

Supplement: S2 Fig — (A) We calculated the average length for equal sets of genes from the UniProt-GOA reference human gene list (orange) and the NHGRI GWAS catalog (yellow) and plotted a histogram of their length distribution. The NHGRI GWAS catalog has much larger average gene loci lengths, therefore in all subsequent analyses we controlled for length. (B) We calculated the average length for equal sets of genes from the UniProt-GOA reference human gene list (orange) and genes from the SNP4Disease GWAS database (yellow). Lists of gene loci selected from the SNP4Disease database are larger than gene loci selected from the reference set. (C) We measured the average gene loci size of reference gene lists as well as the combined disease lists (All) and literature review (L) and GWAS (G) components for all four diseases. The mean and standard deviation of the average loci size of the control lists are shown (black bar, gray box). Compared to PRK, T2D and ALZ, COPD associated loci are the shortest and fall within the expected size of control gene sets. (TIFF) [file pone.0140885.s002.tiff]

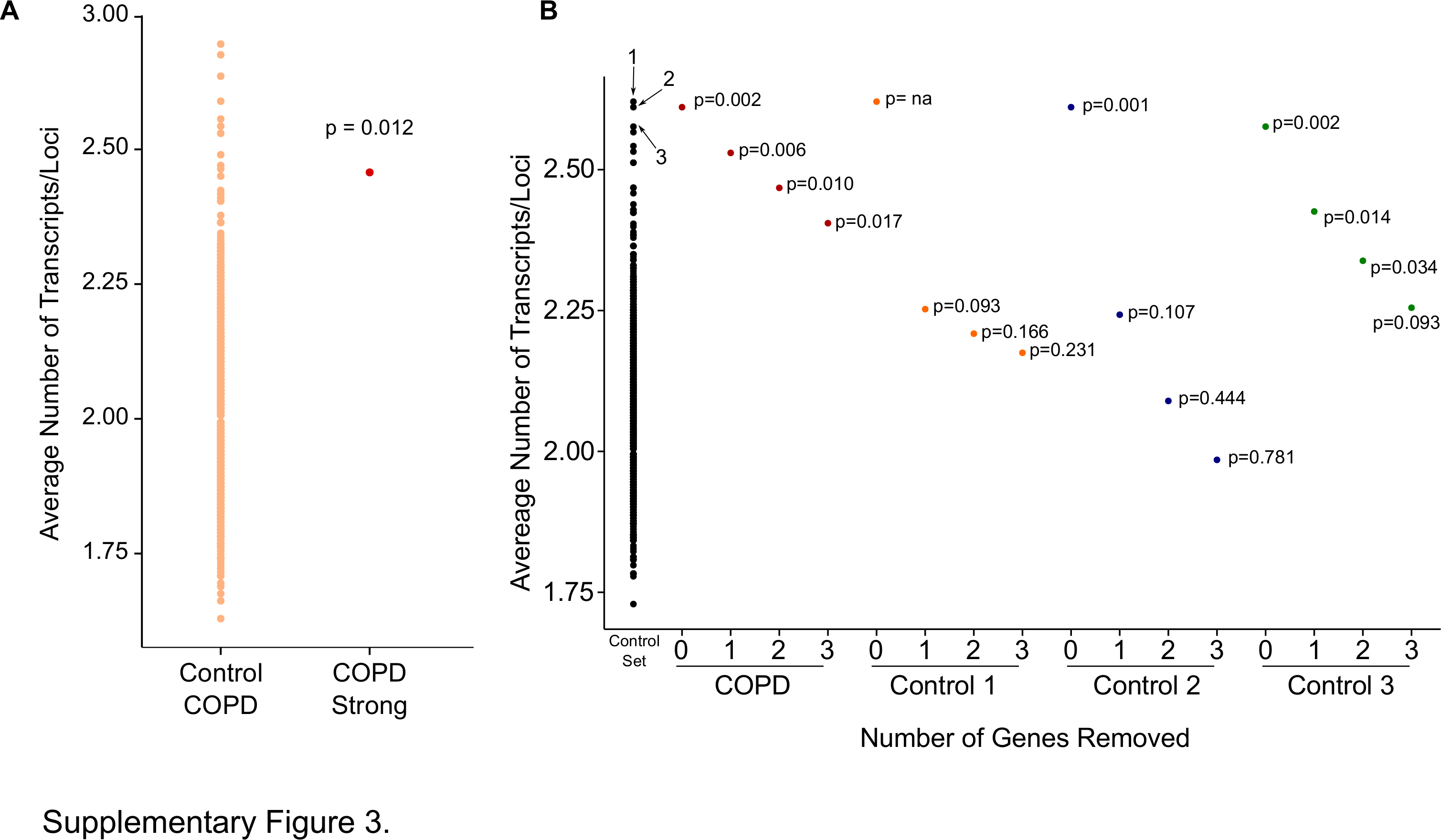

Supplement: S3 Fig — (A) Genes with weak or mixed connection to COPD were removed from the original list and the resulting set was significantly more transcriptionally complex than length-normalized control gene lists. (B) The transcript complexity in the complete COPD list remains significant even when the top transcript producing genes are removed, unlike the top three control lists treated in the same fashion. The p-values are shown in all panels. (TIFF) [file pone.0140885.s003.tiff]

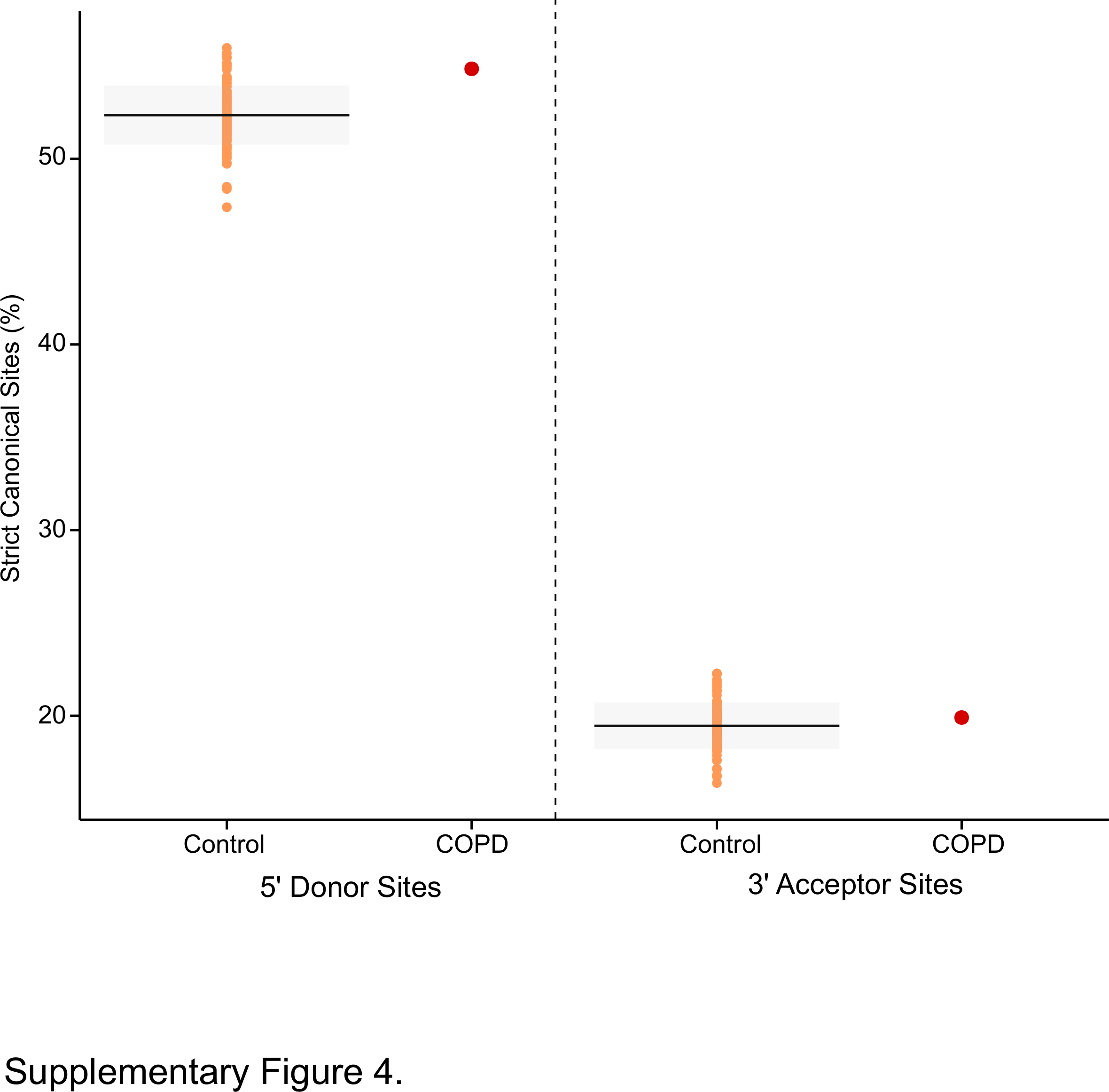

Supplement: S4 Fig — COPD-associated 5’ transcript splice sites contain similar percentages of canonical donor splice sites as control 5’ transcript splice sites (left). The percentage of canonical COPD-associated transcript splice sites at the acceptor site also falls within the expected percentage range of canonical sites, as determined by analyzing the 3’ acceptor splice sites from reference gene sets (right). (TIFF) [file pone.0140885.s004.tiff]

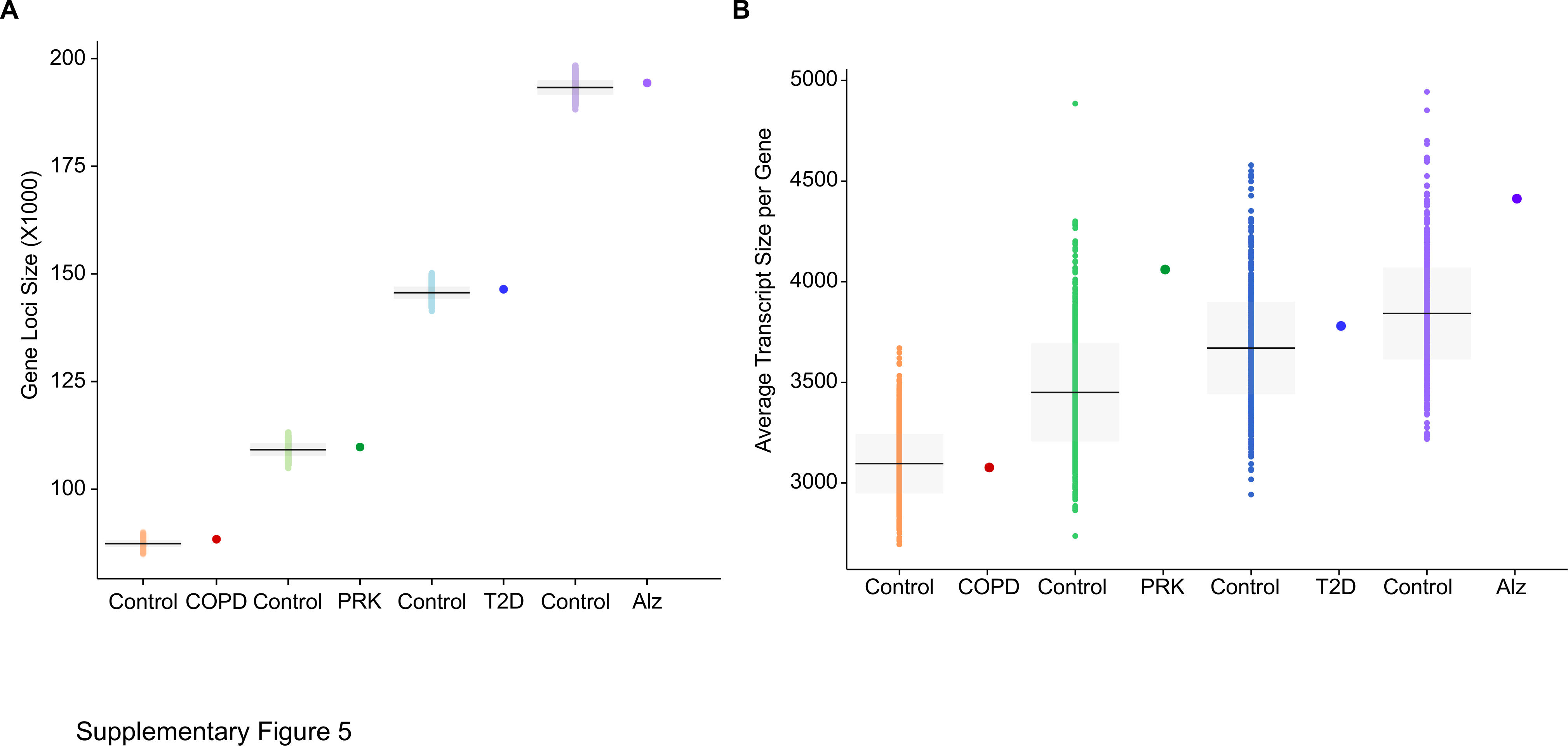

Supplement: S5 Fig — (A) We measured the average gene loci size of normalized reference gene lists and the combined disease lists. The mean and standard deviation of the average loci size of the control lists are shown (black bar, grey box). (B) The average size of COPD-associated mRNAs falls within the range of the average mRNA size produced by length-normalized reference genes. Similar calculations were performed for Parkinson’s Disease, Type 2 Diabetes and Alzheimer’s Disease with controls normalized to each disease list. Disease-associated mRNA average size is within the expected values for all diseases tested. (TIFF) [file pone.0140885.s005.tiff]

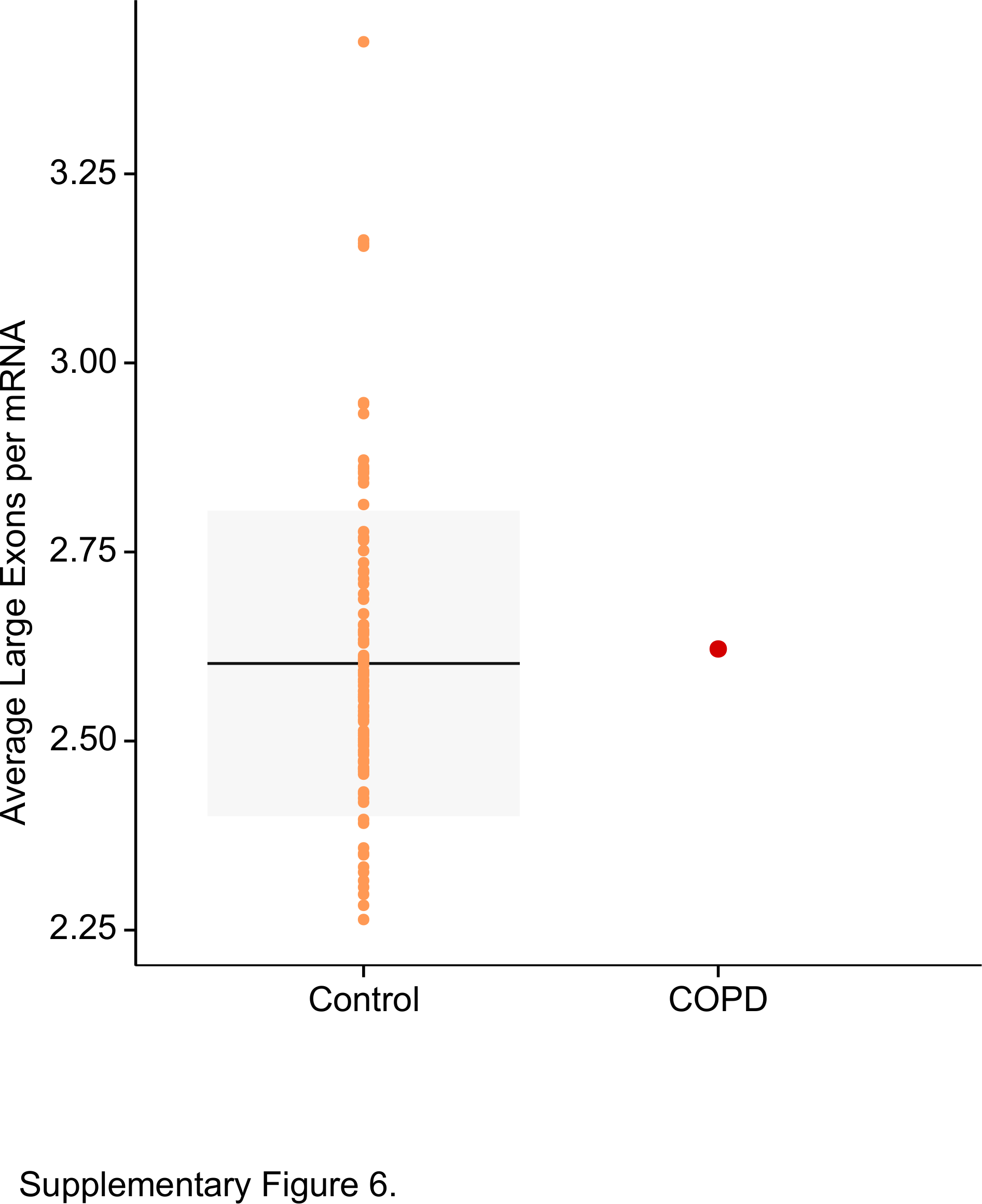

Supplement: S6 Fig — The number of large exons (>200 bps) was calculated for each mRNA and then averaged for all mRNAs produced from each gene list. The average number of large exons per mRNA for the COPD-associated gene list falls within the range of the average number of large exons found in reference gene sets. (TIFF) [file pone.0140885.s006.tiff]

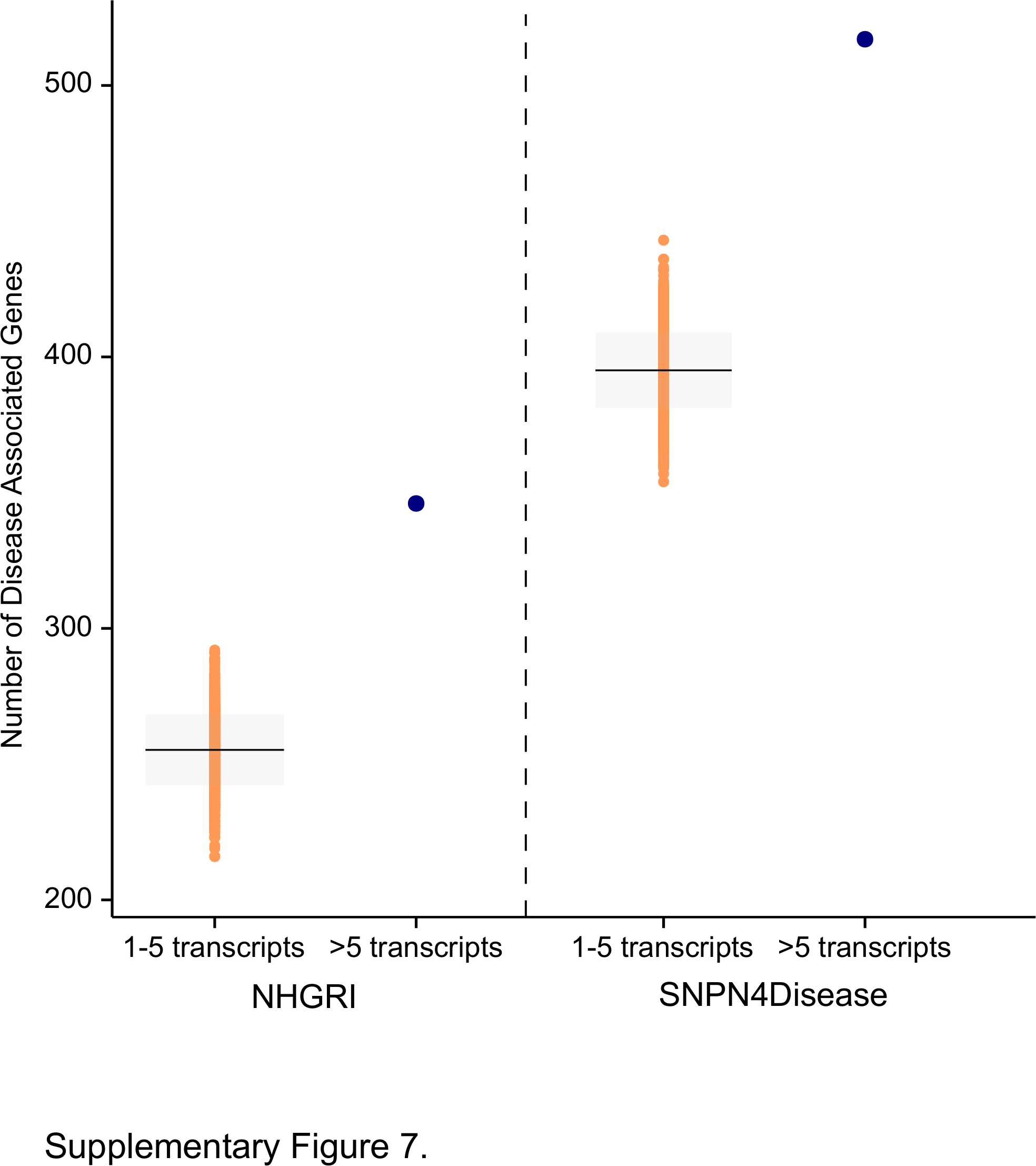

Supplement: S7 Fig — We counted the number of disease-associated genes (identified from public GWAS databases) with 1–5 transcripts compared to those with more than 5 transcripts. Using NHGRI defined disease genes (left), we found that genes with >5 transcripts were significantly enriched for disease-associated genes (p < 0.001). We discovered similar effects when we compared these genes to the SNP4Disease gene list (right), with the gene list of genes with >5 transcripts containing significantly more disease-associated genes (p < 0.001). (TIFF) [file pone.0140885.s007.tiff]

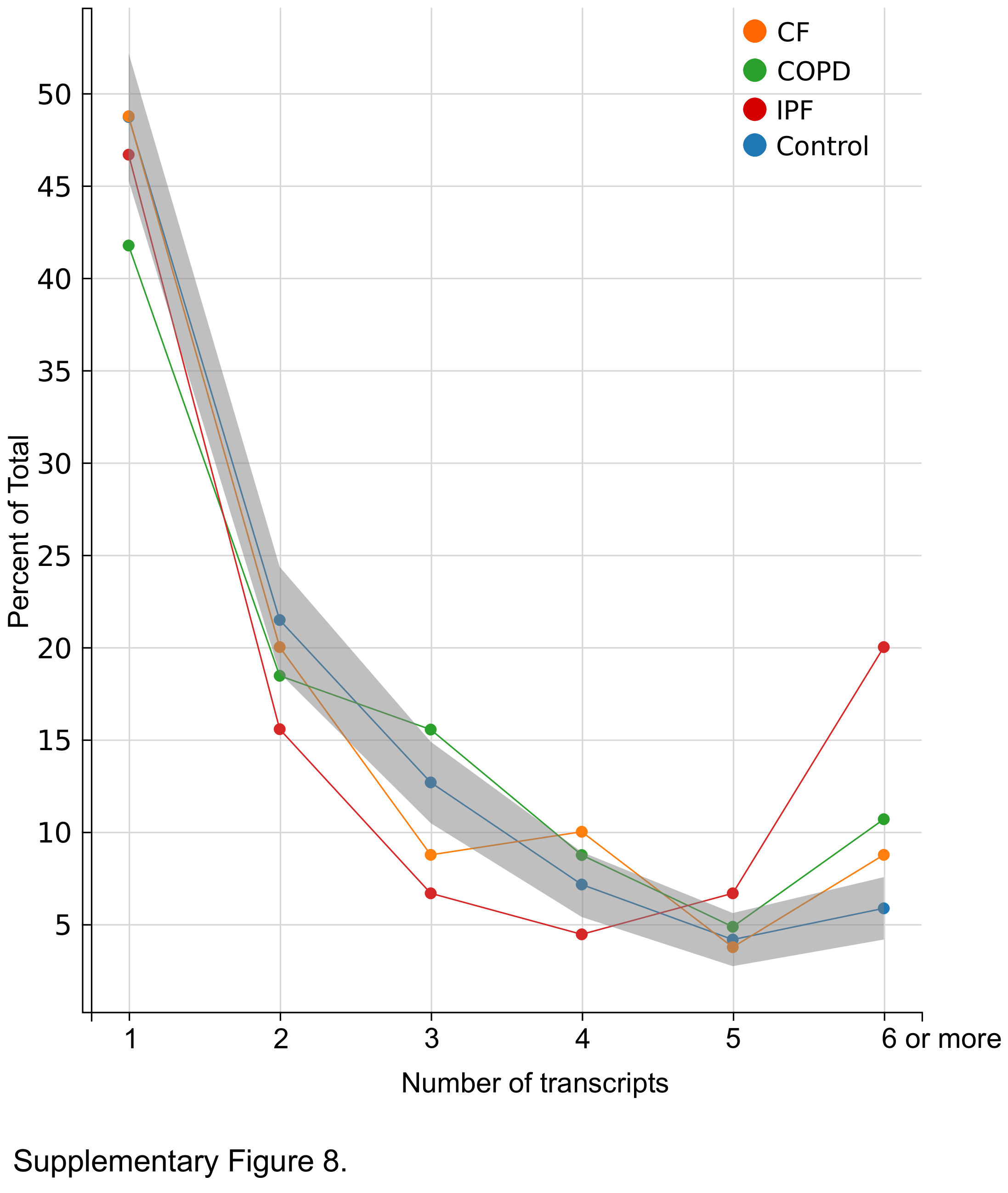

Supplement: S8 Fig — 20% of IPF-associated genes (red) and 10.68% of COPD-associated genes (green) have 6 or more transcripts, while only 8.25% of CF genes (orange) have 6 or more transcripts. The average percentage of total genes with each number of transcripts is shown for 1000 randomized control lists (blue) with the standard deviation shown as a grey ribbon. The low number of genes in the IPF and CF lists impede calculating statistical significance. (TIF) [file pone.0140885.s008.tif]

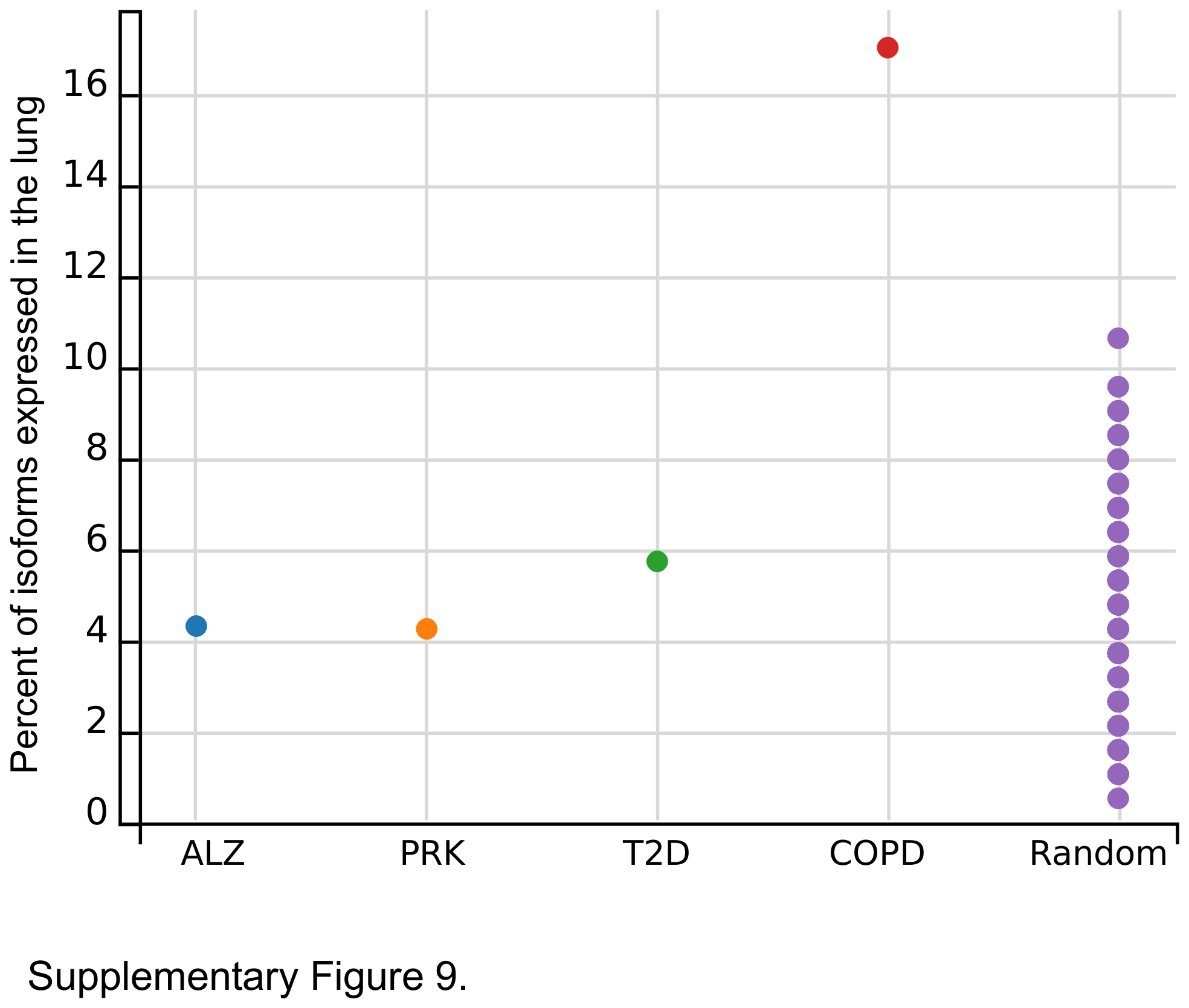

Supplement: S9 Fig — Expression of the most prevalent isoforms of COPD-associated gene transcripts occurred in lung tissue (14.5%) while the percent of isoforms expressed in lung tissue from randomized controls as well as from ALZ, PRK and T2D was much lower (4.82, 4.32, 4.25 and 5.74% respectively). This enrichment in COPD-associated isoforms is significant (p < 0.001). (TIF) [file pone.0140885.s009.tif]

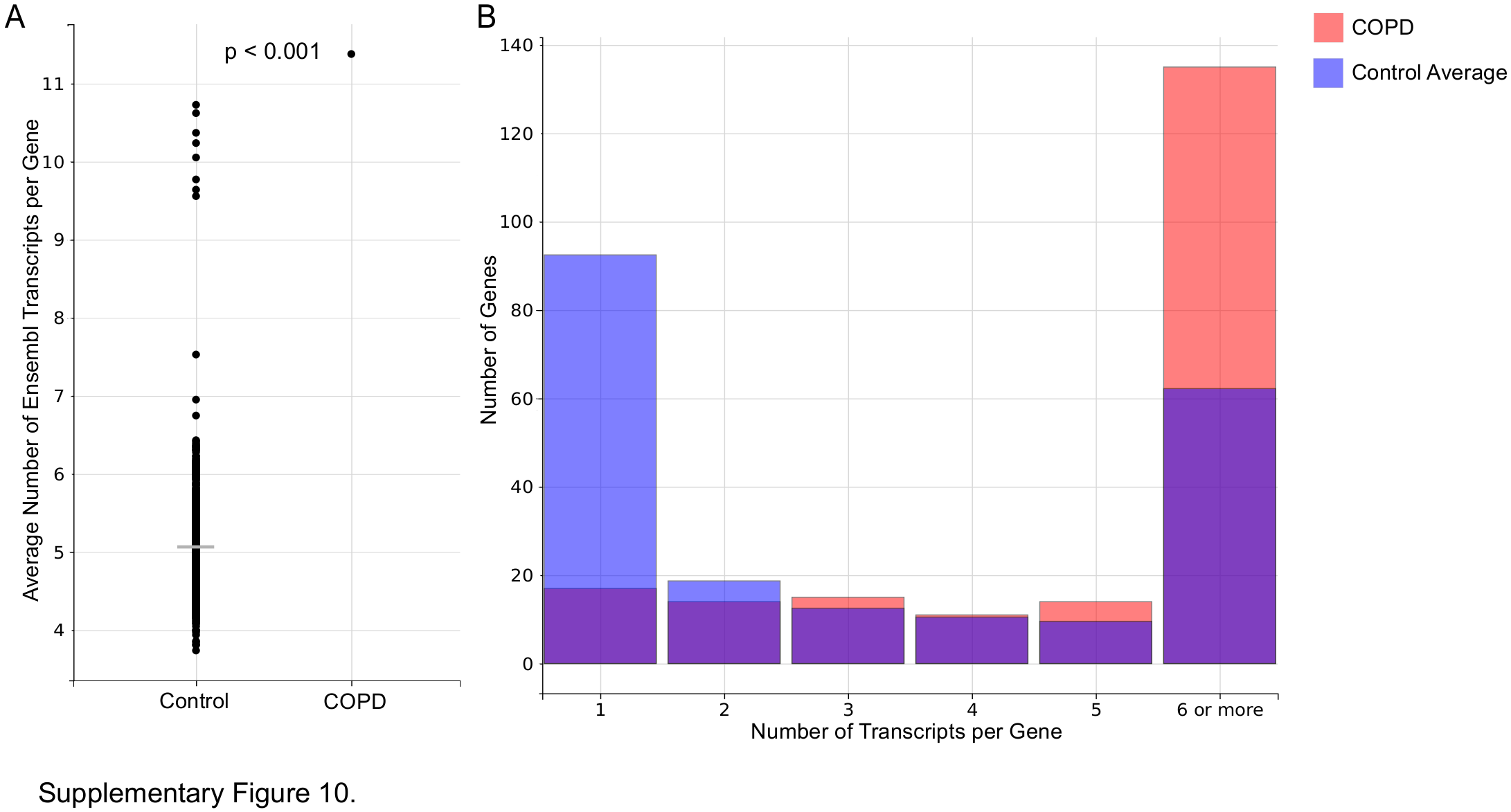

Supplement: S10 Fig — (A) The number of transcripts per gene was calculated using Ensembl defined genes and transcripts for 1000 lists of 206 Ensembl genes as well as the COPD-associated genes. (B) The number of genes with 1,2,3,4,5 and 6 or more transcripts are displayed as an average for 1000 lists of control genes as well as the list of COPD-associated genes. (TIF) [file pone.0140885.s010.tif]
